# Controlled swelling of biomaterial devices for improved antifouling polymer coatings

**DOI:** 10.1038/s41598-023-47192-8

**Published:** 2023-11-15

**Authors:** Alexander H. Jesmer, April S. T. Marple, Ryan G. Wylie

**Affiliations:** 1 Department of Chemistry and Chemical Biology, McMaster University, Hamilton, ON L8S 4M1 Canada; 2 School of Biomedical Engineering, McMaster University, Hamilton, ON L8S 4M1 Canada

**Keywords:** Chemical biology, Chemistry, Biomaterials

## Abstract

Nonspecific interactions between cells and implantable elastomers often leads to failure modes for devices such as catheters, cosmetic and reconstructive implants, and sensors. To reduce these interactions, device surfaces can be coated with hydrophilic polymers, where greater polymer density enhances antifouling properties. Although graft-from coating techniques result in higher density polymer films and lower fouling in controlled settings, simpler graft-to methods show similar results on complex implanted devices, despite limited density. To address the need for improved graft-to methods, we developed Graft then shrink (GtS) where elastomeric materials are temporarily swollen during polymer grafting. Herein, we demonstrate a graft-to based method for poly(oligo(ethylene glycol) methyl ether methacrylate) (pOEGMA) on swollen silicone, GtS, that enhances grafted polymer content and fouling resistance. Total grafted polymer content of pOEGMA on toluene swollen silicone increased over ~ 13 × compared to non-swollen controls, dependent on the degree of silicone swelling. Increases in total grafted polymer within the top 200 µm of the material led to bacterial and mammalian cell adhesion reductions of 75% and 91% respectively, compared to Shrink then Graft (StG) antifouling polymer coated controls. GtS allows for the simple 3D coating of swellable elastomers (e.g., silicone medical devices) with improved antifouling pOEGMA coatings.

## Introduction

Polydimethylsiloxane (PDMS, silicone) is a widely used elastomer for implanted biomaterials^1^ such as catheters, cosmetic and reconstructive implants, and implanted sensors. Nonspecific interactions of these medical device surfaces with bacterial and mammalian cells remains an undesirable occurrence leading to hospital acquired infections^2,3^, degradation of device performance^4^, pain^5^, and implant removal^6^. For example, elastomeric urinary catheters result in two infections per 1000 days of use, making them the most common source of hospital acquired infection^7^ and capsular contraction (i.e., breast implants^8^ and the foreign body response (FBR)) can lead to issues in patient comfort and compromised performance.

To control or eliminate these undesirable interactions, surface topography^9–12^, material stiffness^13^, and antifouling polymer materials have been widely explored^14^. Because polymer coatings can be applied to base materials without changing the underlying properties, they provide a path to device optimization. Thin antifouling coatings of zwitterionic polymers (such as poly(2-methacryloiloxyethyl phosphorylcholine)^15,16^, poly(sulfobetaine methacrylate)^17^ (pSBMA) and poly(carboxybetaine methacrylamide)^18^ (pCB)) and non-charged hydrophilic polyethylene glycol (PEG)^19^ and poly(oligo(ethylene glycol)) methyl ether methacrylate (pOEGMA)^20^ based polymers have shown promise for achieving improved biocompatibility on implantable biomaterials.

PDMS has been functionalized with tethered polymer coatings either by “graft-from” (i.e., surface initiated polymerization)^21–23^, or “graft-to”^24,25^, where pre-synthesized polymers are immobilized onto the surface. Even though graft-from can result in higher polymer densities and thus better antifouling properties^26^, the coating of complex materials (e.g., medical devices) is practically difficult^26^. Indeed, simpler graft-to coatings may perform as well as the in vivo performance of graft-from for complex polymeric devices with the use of end functionalized polymer groups. For example, an experiment comparing the same antifouling polymer coating (pCB) produced by different methods on artificial lungs in extracorporeal circuits in sheep found that using the graft-to method had similar device failure rates as the graft-from method (25–40%)^27^. Thus, efforts to improve graft-to techniques represents a viable strategy for the needed improvement of antifouling polymer coatings.

**Figure 1:**
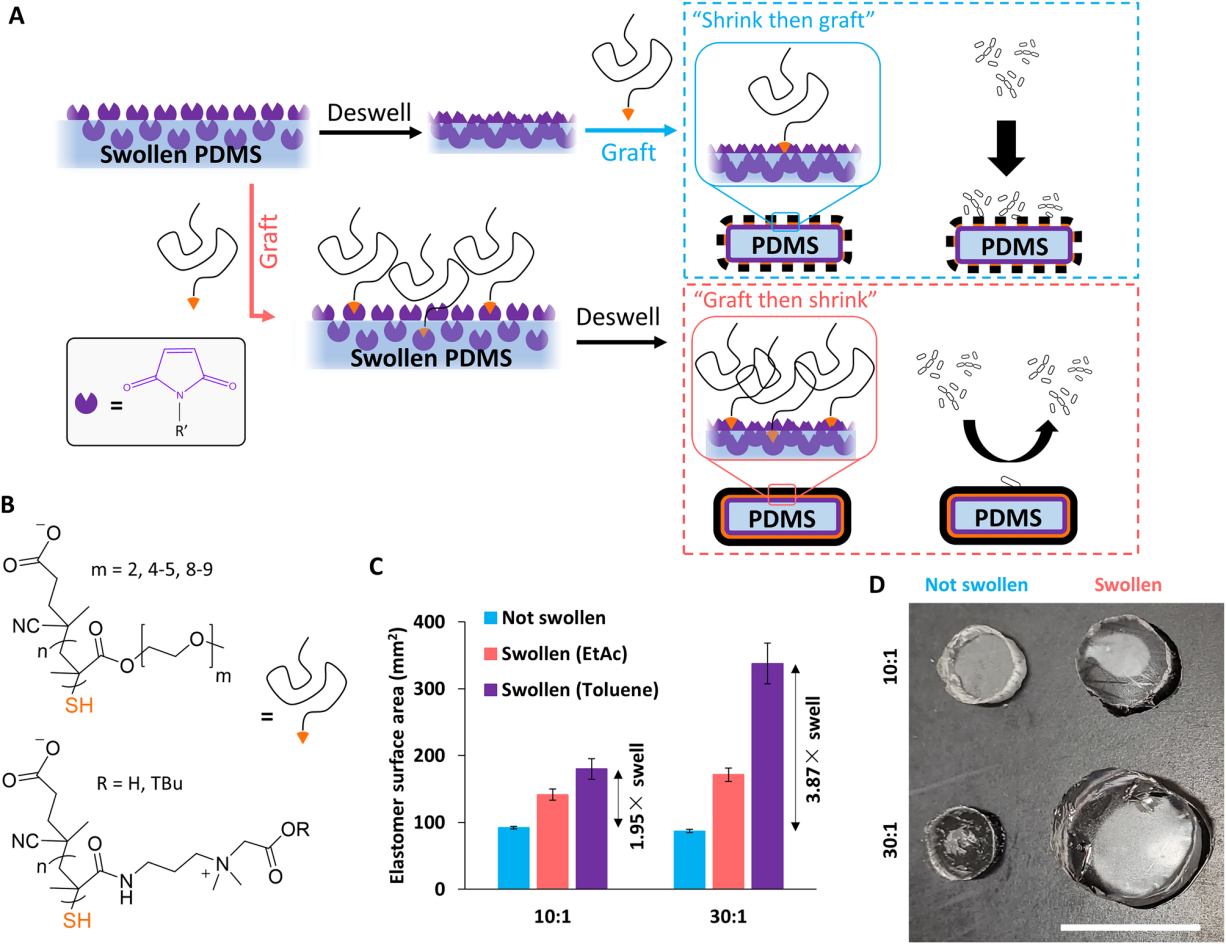
Graft then shrink (GtS) increases grafted polymer content on PDMS. (**A**) Overview schematic of the GtS method using swelled elastomers, where swollen PDMS is functionalized with a hetero-bifunctional NHS-ester maleimide linker (succinimidyl-4-(*N*-maleimidomethyl)cyclohexane-1-carboxylate, SMCC), then either deswelled before grafting thiol terminated polymers (“Shrink then graft” (StG) control) or deswelled after grafting thiol terminated polymers (“GtS”), resulting in improved graft polymer content and antifouling performance. (**B**) Structures of RAFT synthesized thiol terminated pOEGMA 2mer (m = 2), 4mer (m = 4–5) and 8mer (m = 8–9), and pCB-TBu/COOH polymers for grafting to PDMS. (**C**) Controlled swelling for GtS procedures. Swelling ratio with ethyl acetate and toluene of Sylgard™ 184 PDMS with 10:1 (1.95 ×) and 30:1 (3.87 ×) base:crosslinker ratio (mean ± SD, n = 3) by calculation of total surface area using calibrated photographs of top area and thickness, measured in ImageJ. (**D**) Photographs of PDMS in non-swollen and toluene swollen states. Scale bar = 10 mm.

Recently, we developed “Graft then shrink” (GtS) on thermoplastic shrinkable substrates to improve the antifouling performance of graft-to pCB films on gold surfaces. Therein, a thiol terminated polymer layer was grafted onto a gold coated thermoplastic substrate, and then thermally shrunk, producing a microstructured surface with greater grafted polymer content within a defined footprint^28^. Here, we extended the GtS method beyond shrinkable thermoplastics and two-dimensional surfaces to three-dimensional PDMS, a common material for medical devices. Through controlled swelling of PDMS during the grafting of hydrophilic polymers (Fig. 1), we demonstrated increased polymer content and improved antifouling properties towards cells on GtS PDMS devices compared to traditional graft-to methods, providing a simple and effective method to improve graft-to antifouling coatings.

PDMS elastomers were functionalized with maleimide click handles, and a library of 15 thiol terminated pOEGMA and pCB polymers, synthesized via reversible addition-fragmentation chain-transfer polymerization (RAFT), were immobilized to swelled elastomer substrates. The degree of swelling of the elastomer substrate, controlled by combinations of crosslinker density and swelling solvent, was shown to tune total grafted polymer content, with increased swelling yielding greater amounts of grafted polymer. The GtS protocol on elastomers improved the antifouling properties of graft-to pOEGMA coatings towards mammalian macrophages and bacterial *E. coli* by 91% and 75% respectively compared to PDMS modified with the same polymer using the control Shrink then Graft (StG) procedures, where polymers are immobilized to deswelled PDMS. Interestingly, even though pCB content was improved by GtS, no benefit to antifouling properties was observed. Therefore, GtS represents a simple method to improve the already promising graft-to procedures for the modification of PDMS devices with pOEGMA.

## Results and discussion

### GtS with controlled PDMS swelling increases grafted polymer content

**Figure 2:**
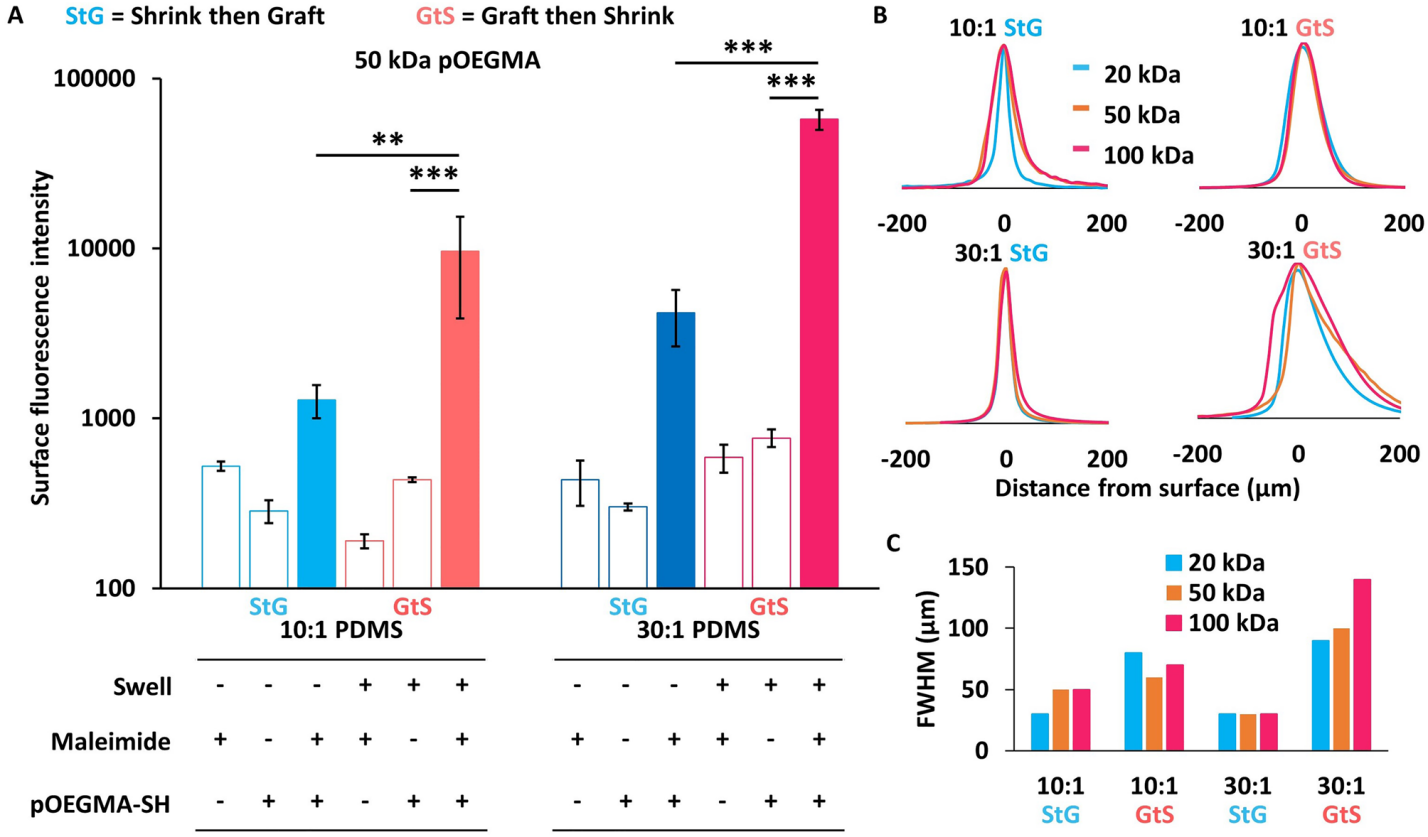
Grafting thiol terminated pOEGMA onto toluene swelled maleimide (SMCC) modified PDMS increases graft polymer content. (**A**) Fluorescence intensity by microscopy of 50 kDa pOEGMA-*co*-fluorescein methacrylate (8mer) copolymers grafted onto PDMS (mean ± SD, n = 3). Unfilled bars represent control elastomers that were either maleimide active with no fluorescent polymer exposure, or pristine PDMS exposed to fluorescent polymer. Filled bars represent elastomers that were both maleimide functional and exposed to thiol terminated fluorescent pOEGMA. Statistical significance was determined using multiple comparisons corrected multiple t-tests, corrected using the Holm-Sidak method, with p values represented as follows, *p* < 0.01 by **, and *p* < 0.001 by ***. (**B**) Confocal microscopy quantification of depth distribution of fluorescently labeled polymer near PDMS surfaces. (**C**) Calculated FWHM of the polymer distributions in (**B**).

Using fluorescent copolymers synthesized with OEGMA monomers, with a 1% feed ratio of fluorescein methacrylate (pOEGMA_f_) and microscopy characterization, it was found that GtS improved grafting efficiency by up to 13 × for pOEGMA_f_ (Fig. 2A) compared to StG control surfaces; polymers grafted onto swollen PDMS (GtS) were compared to control samples where polymers were grafted onto deswelled PDMS (StG). To explore the potential of entrapped non-covalently bound polymer contributing to fluorescence, unreactive PDMS (without maleimides) was exposed to pOEGMA_f_ during GtS procedures, and did not result in significant fluorescent signal, indicating limited polymer entrapment (Fig. 2A, Fig. S1). Moreover, maleimide content on the PDMS surfaces decreased following the polymer immobilization (Fig. S2), though unreacted maleimides remained (10 to 28% unreacted), which were hydrolysed with pH 9.3 borate buffer to avoid conjugations with components in subsequent cell media during nonspecific adhesion assays. Hydrolysis also completes ring opening of the thiol-maleimide adducts, improving long-term stability and avoids potential thiol exchange ^29^. Therefore, GtS through controlled swelling of PDMS can enhance grafted polymer content.

Substrate swelling was first controlled by altering the PDMS base:crosslinker ratio from 30:1 to 10:1 to investigate the influence on grafted polymer content. GtS with swelling in toluene increased pOEGMA_f_ polymer content by 7.5 × on 10:1 PDMS and 13.8 × on 30:1 PDMS compared to respective StG controls. When comparing 30:1 to 10:1, 30:1 resulted in 44.9 × more polymer content, thus greater swelling (30:1 PDMS) provides increased grafted polymer content over less swelling (10:1 PDMS), though even in the StG condition, 30:1 PDMS has greater surface fluorescence than 10:1 PDMS. Confocal microscopy z-stacks showed that all grafted polymer fluorescence was located within the first 200 μm of all PDMS materials studied, with GtS PDMS having broader grafted polymer distributions (full width at half maximum (FWHM) = 60–140 μm) than StG (FWHM = 30–50 μm) (Fig. 2B,C), likely because of polymer penetration into the swollen PDMS.

**Figure 3:**
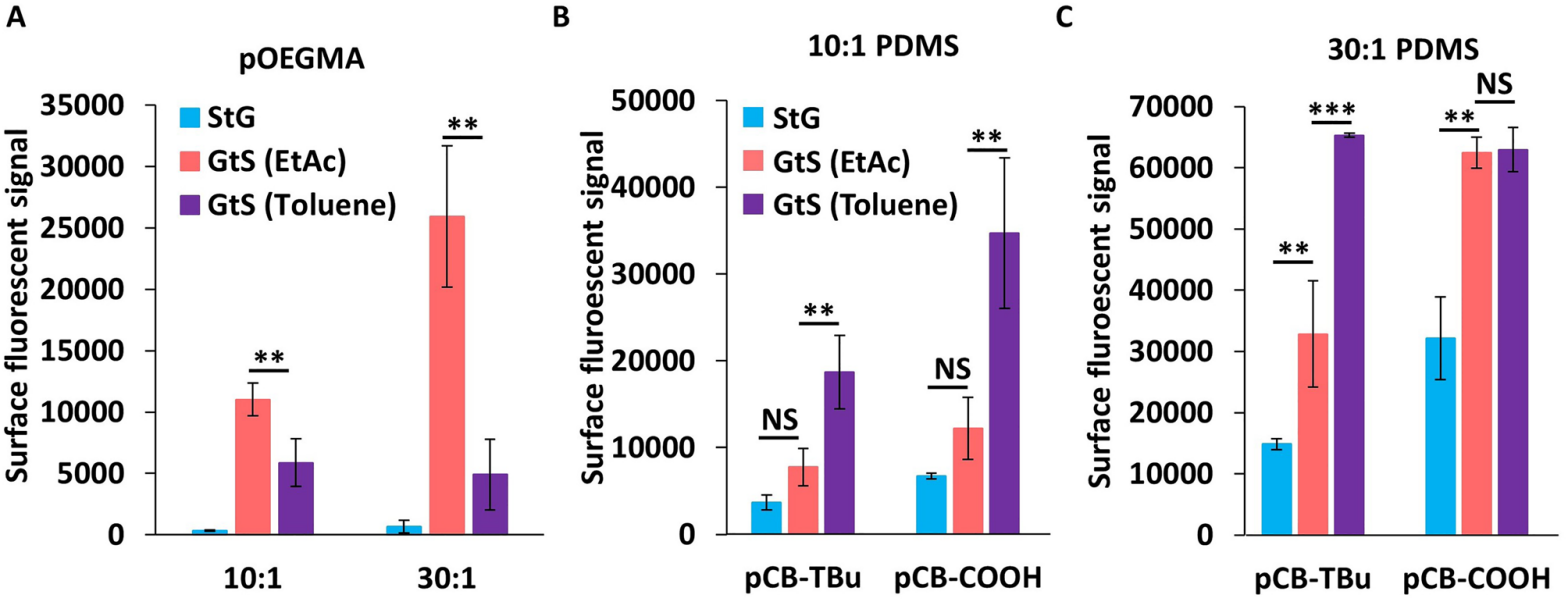
PDMS swelling solvent modulates polymer grafting content. Fluorescence intensity of (**A**) 8mer pOEGMA fluorescent copolymers on both 10:1 and 30:1 PDMS swelled with EtAc or toluene and (**B**) 10:1 and (**C**) 30:1 PDMS swelled in EtAc or toluene functionalized with pCB-COOH and pCB-TBu fluorescent copolymers (mean ± SD, n = 3). Statistical significance was determined using multiple comparisons corrected multiple t-tests, corrected using the Holm-Sidak method, with p values represented as follows, *p* < 0.01 by **, and *p* < 0.001 by ***.

The influence of swelling solvent on polymer grafting efficiency was then investigated with two different solvents to determine the combined effect of swelling ratio and graft polymer solubility (Fig. 3). The two solvents were chosen to have different swelling ratios (*S*, toluene: *S* = 1.31, and ethyl acetate (EtAc): *S* = 1.18) for PDMS (Fig. 1C,D)^35^. Maleimide functional PDMS swollen with either solvent was exposed to pOEGMA_f_ in aqueous 2-(N-Morpholino)ethanesulfonic acid (MES) buffer solutions. EtAc is a good solvent for both PDMS and pOEGMA_f_, which leads to increased grafted polymer content, compared to toluene which is not able to readily solubilize the pOEGMA_f_ but swells PDMS for a greater extent (Fig. 3). The solvent LogP values differ by almost 2, denoting a near 100 × difference in partition (toluene LogP = 2.60, EtAc LogP = 0.65^30^) between water and octanol. Previously it has been shown that pOEGMA based materials can partition between water and EtAc solvent systems, but not between water and toluene systems, partly explaining improved functionalization with EtAc^31^. Therefore, swelling solvent should be optimized for both substrate and grafting polymer.

Oppositely to pOEGMA, pCB polymers showed greater grafted content when conducted with toluene, likely due to the similar solubility of pCB polymers in both swelling solvents. The surface fluorescence intensity of the pCB-COOH_f_ and pCB-TBu_f_ modified elastomers was strongly correlated to swelling ratio as mediated by solvent choice (Fig. 3B,C), providing a route to tailor grafted polymer content independent of crosslinker density so that elastomer properties such as stiffness can be partially tailored outside of swelling degree. For example, 10:1 PDMS swelled with toluene and 30:1 PDMS swelled with EtAc have similar swelling ratios (1.95 and 1.96 respectively) but the 30:1 elastomer had 76% more surface fluorescence upon grafting fluorescent pCB-TBu_f_. Therefore, PDMS swelling for GtS applications can be controlled by solvent swelling, crosslinker density or a combination thereof. Further, zwitterionic pCB-COOH_f_ resulted in more grafted polymer than the positively charged, and more hydrophobic, pCB-Tbu_f_, potentially due to electrostatic repulsion between the polycations (Fig. 3). It should be noted that the pCB-TBu_f_ and pCB-COOH_f_ fluorescent content was identical as both polymers were sourced from the same batch; pCB-COOH_f_ was produced by deprotecting pCB-TBu_f_ under acid conditions (Fig. S3). As most graft-to procedures, polymer–polymer interactions within the coating influence grafting density.

The effect of buffer composition and salt concentration on grafted polymer content were tested using zwitterionic pCB-COOH_f_ given the effects salts exhibit on zwitterion hydration from the antipolyelectrolyte effect^32^. Two grafting buffer compositions were compared, MES and guanidine HCl (GHCl), at four concentrations between 1 and 1000 mм. MES has been previously used in similar applications, and GHCl is a strong chaotrope and guanidine salts have been shown to interact with amide bonds present in the methacrylamide backbone of the pCB-COOH_f_^33^. Interestingly, the incorporation of specific GHCl concentrations enhanced the grafting of pCB-COOH_f_, with 10 mм GHCl resulting in the greatest grafting degree, whereas MES increased grafting content with increasing concentrations (Fig. S4). Absorbance of PDMS and the loss of fluorescence from the grafting solution was used to quantify the grafting degree of the pCB-COOH_f_ copolymer; the high grafting density on the PDMS resulted in fluorescence quenching^34,35^ (Fig. S4). Absorbance values at 502 nm were used to quantify polymer content in lieu of fluorescence, where 10 mм GHCl values were the highest overall, with intense absorbance due to fluorescein methacrylate content observed.

### Characterization of biological fouling properties

**Figure 4:**
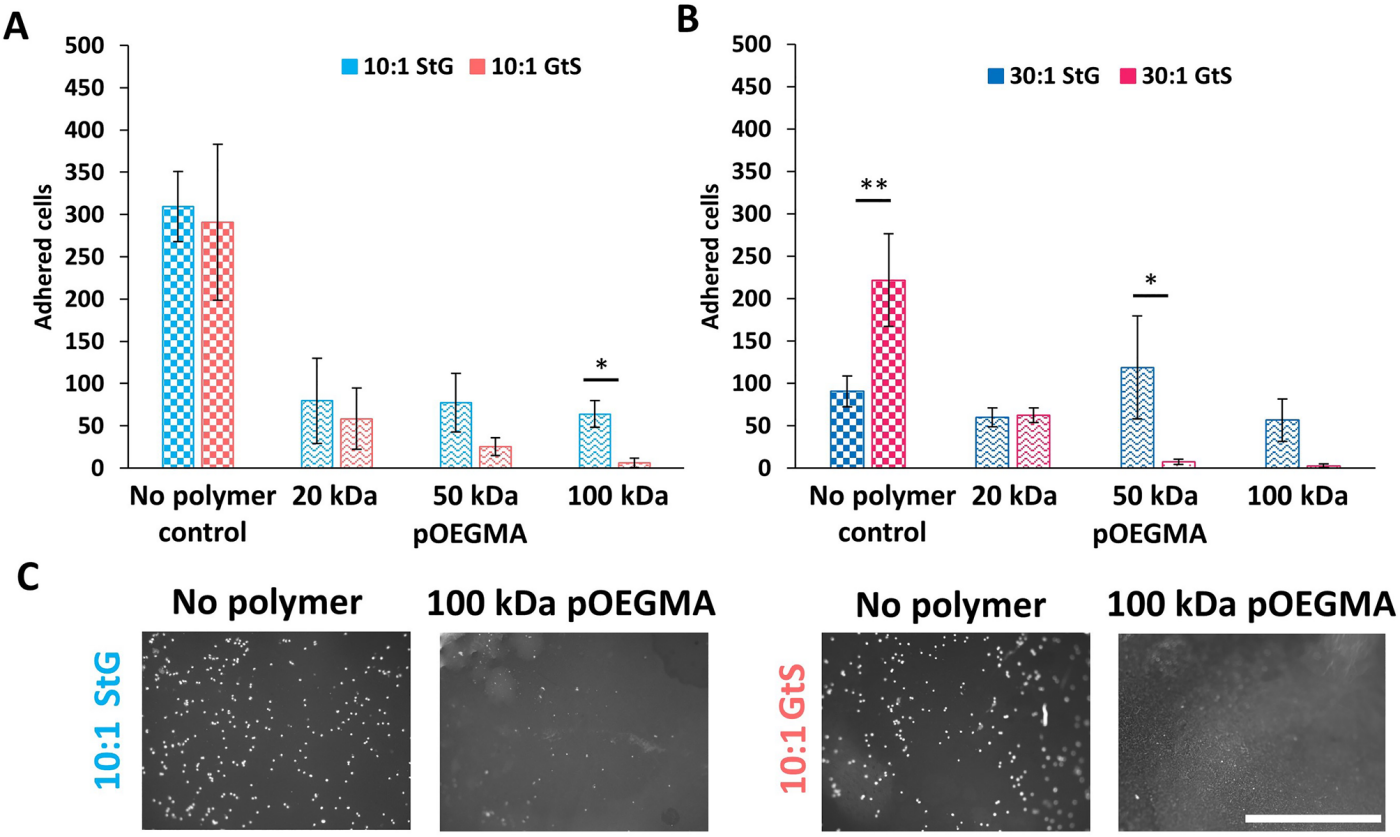
GtS with high M_w_ and hydrophilic pOEGMA improves antifouling properties. (**A**, **B**) Cell adhesion of Raw 264.7 macrophages on 10:1 and 30:1 PDMS modified with pOEGMA (mean ± SD, n = 3). No polymer control refers to PDMS surfaces functionalized with SMCC and hydrolyzed before exposure to cells. Statistical significance was determined using multiple comparisons corrected multiple t-tests, corrected using the Holm-Sidak method, with p values represented as follows, *p* < 0.05 by *, and *p* < 0.01 by **. (**C**) Representative fluorescence micrographs of cell adhesion on 10:1 PDMS modified with 100 kDa pOEGMA. Scale bar = 1000 µm. Representative fluorescence micrographs of all other conditions are shown in Fig. S8.

For implanted medical devices, macrophage adhesion and activation are important parameters and metrics for relative FBRs^36^, and specifically macrophage adhesion to silicones is related to negative clinical outcomes^37^ making them an important model cell type for antifouling performance. The nonspecific adhesion of RAW 264.7 macrophages to functionalized PDMS was therefore characterized by fluorescence microscopy. Interestingly, GtS improved the antifouling properties of pOEGMA towards macrophages but not pCB (see section "Further discussion" for further discussion and Fig. S6), pOEGMA was therefore selected for further study. In all pOEGMA conditions GtS was equally or more antifouling than StG, with 100 kDa pOEGMA on 10:1 PDMS reducing adhesion by 98% compared to hydrolyzed succinimidyl-4-(N-maleimidomethyl)cyclohexane-1-carboxylate (SMCC) PDMS controls (6 ± 5 and 291 ± 92 respectively) and 91% compared to the StG condition (64 ± 16), with similar improvements on the 30:1 PDMS (50 kDa pOEGMA StG = 118 ± 61, and GtS = 7 ± 3, 100 kDa pOEGMA StG = 57 ± 25, and GtS = 3 ± 2) (Fig. 4). Therefore, GtS is suited for the functionalization of PDMS using pOEGMA polymers, and can improve fouling resistance towards macrophages by up to 98%.

Fouling resistance was found to be M_w_ dependent, with pOEGMA being most antifouling at the highest studied M_w_s in the GtS condition (in agreement with bacterial resistance on pOEGMA presented below), potentially due to a thicker layer being produced at higher M_w_s, which provides improved antifouling^38,39^. We have previously seen resistance towards macrophage adhesion to be dependant on polymer molecular weight (M_w_) for wrinkled gold GtS surfaces, but no correlation on flat or StG was seen^28^. Here, pOEGMA based polymers on both 10:1 and 30:1 PDMS show similar M_w_ dependence on GtS conditions and not on StG surfaces, as expected from previous studies.

Applying the GtS procedure to pOEGMAs with shorter 2 and 4–5 repeat unit OEG side chains, with cLogp values greater than 0 (0.89 and 0.36 respectively) did not improve their resistance to cell adhesion. On the highly swelling 30:1 PDMS, 2mer and 4mer pOEGMA reverse the effect of the GtS condition and have significantly increased cell adhesion, of 359 and 403% respectively, compared to StG (Fig. S7). 10:1 PDMS produced insignificant differences on 2mer and 4mer surfaces between GtS and StG, indicating insufficient polymer differences to influence fouling. GtS can increase cell adhesion depending on the properties of the polymer to be grafted.

#### Nonspecific bacterial transfer and detachment

**Figure 5:**
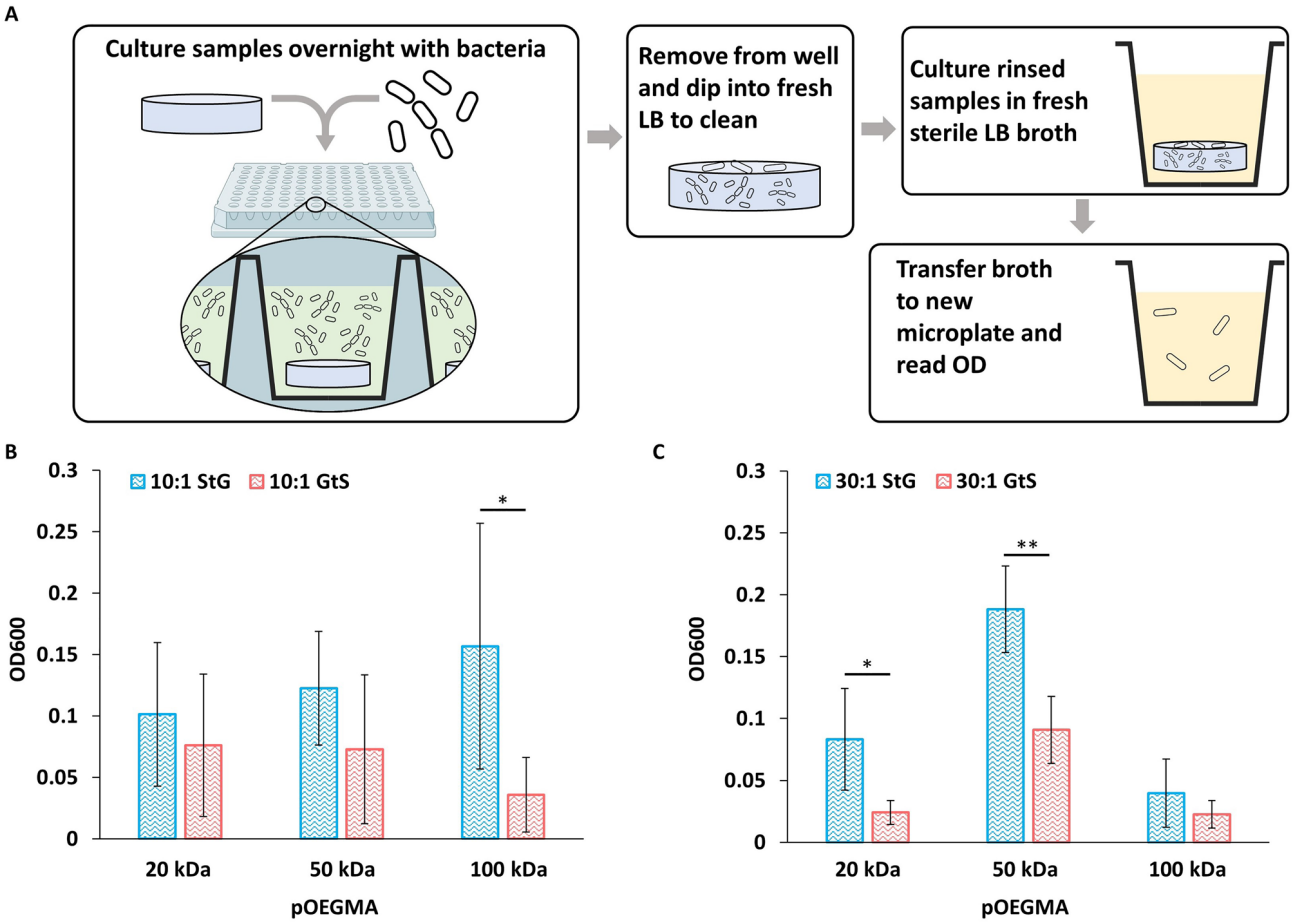
GtS improves the resistance of pOEGMA modified elastomers towards *E. coli* bacterial transfer. Live bacterial transfer was characterized by (**A**) culturing the elastomers in *E. coli* suspensions overnight and then gently rinsing the elastomers with sterile LB broth and incubating them in fresh LB broth overnight, allowing adhered bacteria to proliferate. OD600 values of the LB broth following overnight incubation with bacteria exposed (**B**) 10:1 and (**C**) 30:1 elastomers modified with pOEGMA and pCB-COOH was then measured (mean ± SD, n = 4). Statistical significance was determined using multiple comparisons corrected multiple t-tests, corrected using the Holm-Sidak method, with p values represented as follows, *p* < 0.05 by *, and *p* < 0.01 by **.

Given the prevalence of silicones as urinary catheters and that *E. coli* is the most common cause of catheter associated urinary tract infections^40^, *E. coli* was selected as a model organism for bacterial assays. Similar to nonspecific macrophage adhesion, the GtS protocol with 8mer pOEGMA coatings consistently reduced the transfer and proliferation of live *E. coli*. Because the polymer materials used are not antibacterial but are cell repellent, we measured bacterial transfer (Fig. 5A) in a setup to mimic how non-antibacterial medical devices can transport bacteria into hosts during clinical use and implantation. To detect small degrees of live bacterial binding, PDMS materials were exposed to bacteria under orbital shaking, then gently dipped into three sequential sterile LB broth wash containers to remove unbound bacteria and finally incubated overnight in sterile LB growth media before bacterial detection by optical density at 600 nm (OD600). 100 kDa 8mer pOEGMA GtS on 10:1 PDMS (OD600 = 0.04 ± 0.03) showed the best improvement at resisting *E. coli* transfer and proliferation compared to the corresponding StG condition (OD600 = 0.16 ± 0.1; Fig. 5). For live bacterial transfer, no consistent trend was observed between 10:1 and 30:1 PDMS, with increasing pOEGMA M_w_ improving antifouling properties for 10:1 PDMS but not 30:1. In the bacterial transfer test, GtS was shown to improve the resistance to bacteria transfer when compared to StG controls for most pOEGMAs studied.

### Physical characterization of modified elastomers

**Figure 6:**
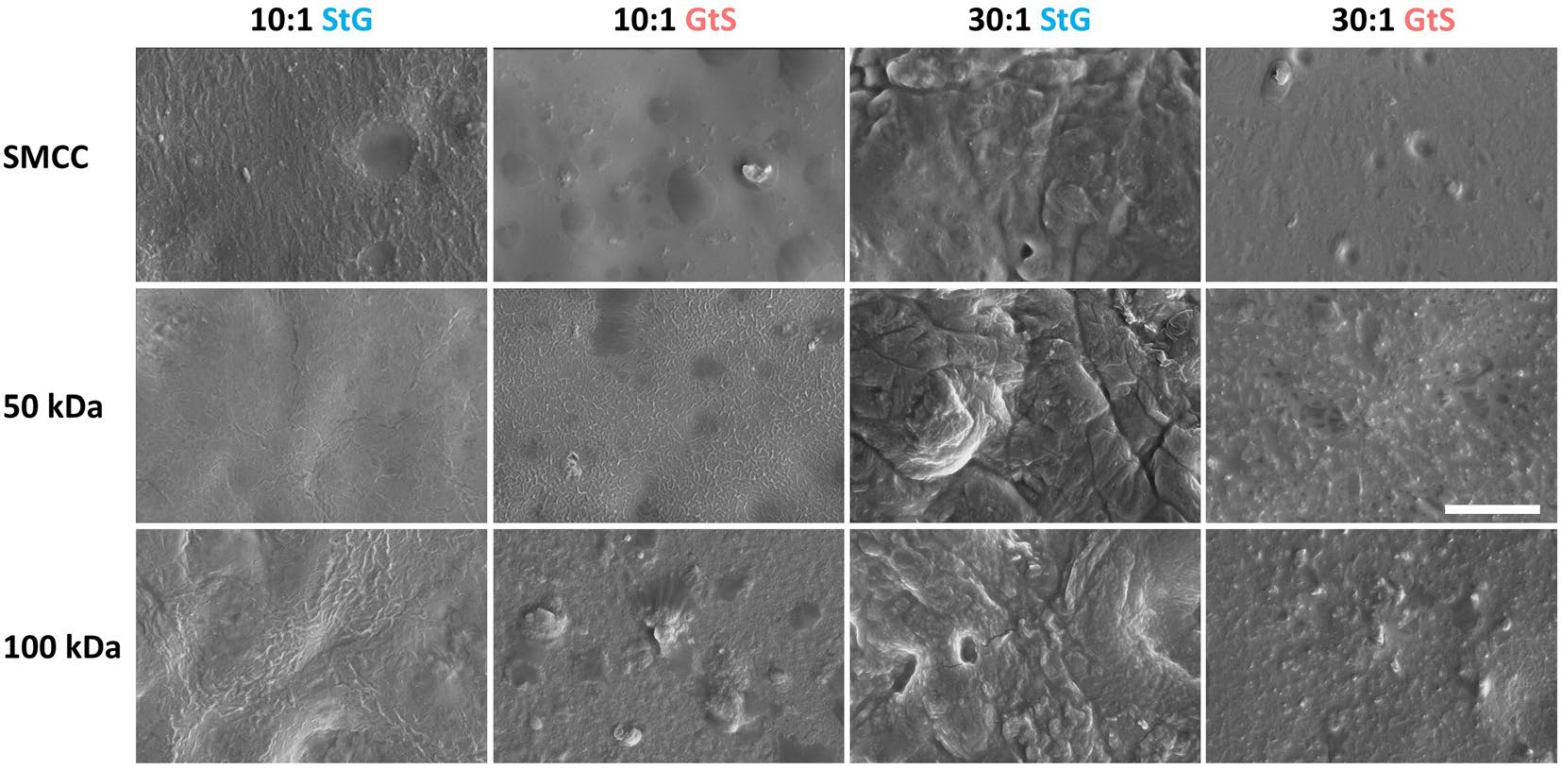
Elastomer swell state during graft procedure, and surface grafted polymer, modifies surface structure. SEM micrographs of 10:1 and 30:1 PDMS surfaces coated with SMCC, 50 kDa or 100 kDa pOEGMA. SMCC no polymer control surfaces are functionalized with SMCC, and then incubated in MES buffer either not-swelled (StG) or swelled with toluene (GtS). Scale bar = 4 µm.

Because the GtS procedure may result in surface topography differences, elastomer surface morphology was investigated with scanning electron microscopy to study differences between base composition, swell state during grafting, and presence of grafted pOEGMA (Fig. 6). On non-polymer coated surfaces (i.e. functionalized only with APTES and SMCC, and then exposed to MES for 4 d with no pOEGMA) micrographs revealed a qualitatively smoother surface on both 10:1 and 30:1 PDMS when soaked in MES in the swelled state than the deswelled state. On 30:1 materials, these larger features were apparent on StG surfaces in pOEGMA coated conditions as well, potentially influencing cell adhesion results. On the 10:1 and 30:1 GtS surfaces, both 50 and 100 kDa pOEGMA coated surfaces had wrinkled and rough surface features of ~ 70 to 160 nm that were not apparent on the MES surfaces, with both 100 kDa modified GtS surfaces having several larger features within the field of view.

Because water contact angle is often used to predict antifouling properties of surfaces by comparing hydrophilicity, we compared pOEGMA of varying OEG side chain length (2mer, 4mer, and 8mer) and pCB-COOH grafted before and after deswelling. Although, the swelling procedure within GtS will enhance PDMS hydrophobic recovery, making WCA comparisons difficult. For nearly all polymer compositions, M_w_s and base ratios, StG and GtS surfaces had statistically similar hydrophilicities, with pristine, unmodified base PDMS having contact angles of 110° ± 6 and 118° ± 7 for 10:1 and 30:1 respectively.

**Figure 7:**
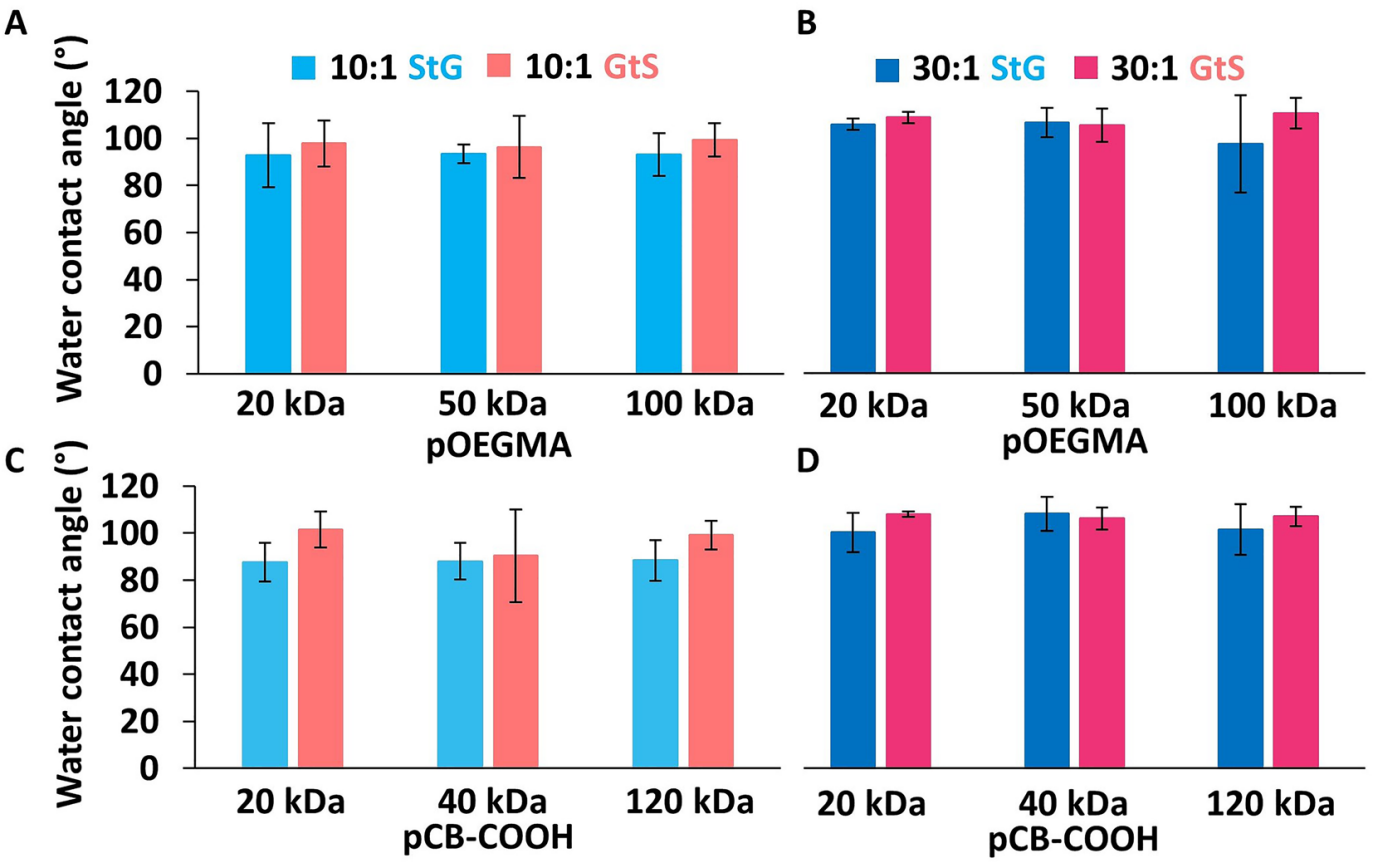
GtS procedures on PDMS do not modify hydrophilicity by WCA. (**A**)WCAs of 3 μL droplets of Milli-Q water on pOEGMA modified (**A**) 10:1 and (**B**) 30:1 PDMS and pCB-COOH modified (**C**) 10:1 and (**D**) 30:1 PDMS elastomers. The lack of WCA changes is likely due to PDMS hydrophobic recovery from the solvent swelling procedure in GtS. Mean ± SD, n = 4.

In the water contact angle comparisons, 2mer modified elastomers were the lone exception with GtS elastomers displaying increased hydrophilicity compared to StG. Interestingly, the traditionally more hydrophobic 2mer modified PDMS also showed the lowest absolute water contact angle at 67° (Figs. 7 and S9), despite being the most hydrophobic monomer (cLogP = 0.89). Water contact angles (WCA) of graft-from pOEGMA on PDMS have been reported between 54° and 71° depending on layer thickness, appreciably lower than most of the values for pOEGMAs reported here, indicating polymer grafting was either not as dense or as thick as traditional graft-from procedures^41^, even though GtS enhanced antifouling properties towards macrophages and bacteria for the 8mer pOEGMA. Because of the interesting WCA using MilliQ water, we next investigated contact angles in cell culture media used in the nonspecific macrophage adhesion assay. The contact angle of cell culture media to 8mer pOEGMA surfaces were similar to that of MilliQ water, with all values between 89° and 98°, except for the 10:1 StG condition where contact angles of cell media were lower than those of water, at 67° to 81° (Fig. S10).

The change in WCA between the two conditions is influenced by two variables, the amount of grafted hydrophilic polymer, and the accelerated hydrophobic recovery of the PDMS surface due to the extended solvent swelling in the GtS state. In the GtS procedure PDMS was swollen during the 4 d polymer grafting where StG was not, which can lead to differences in PDMS hydrophobic recovery. To validate if the differences in swelling procedure influences the WCA, we prepared SMCC modified PDMS by GtS and StG without grafting polymers (i.e., exposed swollen and non-swollen SMCC modified elastomers to MES buffer for 4 d). The SMCC modified StG surface had lower WCAs (10:1 = 80° ± 10, 30:1 = 105° ± 3) than SMCC modified GtS surfaces (10:1 = 96° ± 4, 30:1 = 107° ± 1), demonstrating that both lower crosslink density and swelling increases hydrophobic recovery. Even though GtS results in greater polymer immobilization and improved antifouling properties towards cells (Figs. 2 and 4), GtS results in higher or similar WCAs for hydrophilic polymers because of accelerated hydrophobic PDMS recovery from solvent swelling compared to StG. When then grafted a pCB-COOH polymer layer that had amine functionality added through copolymerization with N-(3-aminopropyl) methacrylamide and was crosslinked via incubation with 0.1 M EDC and 0.1 M NHS before deswelling to reduce hydrophobic recovery, the WCA was decreased (54° to 75°) compared to the non-crosslinked graft layers above (Fig. S11). Further evidence of hydrophobic recovery, or surface adaptation, was found when advancing and receding water contact angles were measured of the most antifouling 100 kDa 8mer pOEGMA surfaces, with receding contact angles of 17 to 42°, and significant hysteresis of between 52 and 74° apparent on all tested materials (Fig. S11C). For hydrophobic polymers (i.e., pOEGMA 2mer), GtS decreased WCAs which may be due to hydrophobic POEGMA-POEGMA interactions (lower critical solution temperature of 26 °C for 2mer) limiting rearrangement at the surface.

### Further discussion

The efficiency of the GtS method is maximized when the properties of the graft polymer—swollen elastomer system are chosen to achieve a solvated reaction environment and a high degree of swelling. The total grafted polymer content was highest when using neutral high M_w_ pOEGMAs, coupled with a low crosslink density elastomer, which maximizes swelling, that is swollen with a solvent that also solubilizes the graft polymer. In this case, considering LogP, allowed the solvent to be matched for swelling purposes and maximizing the graft polymer partitioning onto the swollen surface.

The two polymer types investigated here represent two separate classes of hydrophilic antifouling polymer, uncharged, and neutral charged (i.e., zwitterionic). Both pCB and pOEGMA prevent protein adsorption via strong interactions with a water shell, but the branched pOEGMA also repels protein through steric repulsion due to the long (M_n_ = 500 Da) OEG side chains. These differences in antifouling mechanisms, and the difference in cLogP between the constituent monomers (8-9mer OEGMA = − 0.34, CB = − 6.88) may make OEGMA based polymers more amenable to the Graft then Shrink method on hydrophobic PDMS and other medically relevant elastomers. Moreover, because GtS distributed the polymer within the first ~ 200 µm, the larger and branched pOEGMAs may improve antifouling activity at the surface through steric repulsion mechanisms. Further, the thermoresponsive properties of OEGMA based polymers have also recently been used to control drug release of antibacterial molecules at physiologic temperatures from polymer coatings on implants^42^.

The GtS method for swellable elastomeric substrates could be extended to other commonly used medical polymers such as polyurethane or polyvinylchloride^43^, especially those that have tunable crosslink densities and swelling ratios, and that are highly swellable with solvents that also solubilize the graft polymer, similar to the PDMS-EtAc-pOEGMA system. The wide range of relevant swellable materials, growing number of ways to fabricate antifouling polymer films via graft-to, and recent indications that the graft-to method can perform as well as graft-from in medical applications means that the GtS method represents a path to improve the simpler and similarly performing technique. The method could also be used on PDMS with higher swelling ratios such as 60:1 Sylgard™ 184^44,45^. Grafting of polymers onto swelled elastomers could also be performed using physicochemical methods such as 3,4-dihydroxyphenylalanine (DOPA) anchoring rather than click reactions to expand potential materials for coating^46^. Though click reactions, especially thiol ene reactions, are well suited for GtS and have been previously explored for the modification of biomaterial surfaces^47^, due to the simple production of thiol terminated polymers and the catalyst free mild reaction conditions^48^. Finally, the growing array of click reactions allow for the method to be extended to allow for multiple types of polymers to be patterned at once, before shrinking the material to improve final fidelity. Therefore, GtS has the potential to be widely applied to improve medical devices and implants.

## Conclusions

GtS offers a simple method to improve the antifouling properties of polymer coated silicone devices and graft-to techniques, demonstrating an improvement of 91% against mammalian cell adhesion over conventional graft-to methods when used with 8mer POEGMA. The GtS technique was applied to two formulations of commercially available PDMS with multiple antifouling homo and copolymers of various compositions demonstrating the importance of swelling conditions and grafting polymer selection to improve antifouling properties of PDMS devices using GtS. The degree of swelling of the elastomeric substrate can tune total polymer grafting, either through crosslink density or through swelling solvent choice. GtS on stiffer 10:1 material with higher M_w_ pOEGMA (100 kDa) had the best overall results, reducing macrophage adhesion by 98% compared to hydrolyzed SMCC controls and 91% compared to 10:1 PDMS coated using StG, potentially due to the grafted pOEGMA penetrating less deeply into the elastomer compared to the 30:1 PDMS, leading to a higher concentration near the surface of the material. Although, GtS did not improve the antifouling properties for pCB even though polymer content was increased, indicating that polymer and swelling procedures must be carefully chosen. With appropriate swelling solvent and polymer choice, GtS offers a method to both increase polymer coating content and improve antifouling properties for graft-to procedures on PDMS.

## Materials and methods

### Materials

N-[3-(dimethylamino)propyl]methacrylamide, tert-butyl bromoacetate, trifluoroacetic acid (TFA), 4-Cyano-4-(phenylcarbonothioylthio)pentanoic acid, 4,4’-azobis(4-cyanovaleric acid), sodium hydroxide, 2-(N-morpholino)ethanesulfonic acid sodium salt (MES), ethanol, sodium acetate, and Oligo(ethylene glycol) methyl ether methacrylate (M_n_ = 186, 300, and 500), fluorescein methacrylate, fluorescein maleimide assay kit, guanidine hydrochloride, (3-aminopropyl)triethoxy silane (APTES), acetonitrile, N-(3-Dimethylaminopropyl)-N′-ethylcarbodiimide hydrochloride, N-hydroxysuccinimide (NHS), Tris(2-carboxyethyl)phosphine hydrochloride (TCEP), and ethyl acetate were purchased from Sigma Aldrich (Oakville, ON, Canada). Fetal bovine serum (FBS), Calcein AM, Hoescht, and Sylgard 184 elastomer kit was obtained from Thermo Fisher Scientific (Burlington, ON, Canada). LB broth was purchased from Bioshop Canada (Burlington, ON, Canada). SMCC was donated by Todd Hoare from McMaster University (Hamilton, ON, Canada). Phosphate buffered saline (PBS) at pH 7.4 contained 10 mм sodium phosphate and 137 mм NaCl.

### PDMS elastomer preparation and swelling

PDMS was prepared using a Sylgard™ 184 elastomer kit, with ratios of 10:1 and 30:1 base to crosslinker. Elastomers were mixed, degassed, and then cured for 30 min at 80 °C. Discs of 3 mm thickness and 6 mm diameter were punched out using a leather punch. Discs were then extracted with toluene 4 times to remove uncured free PDMS. Finally, discs were deswelled and stored at room temperature until use.

### *Tert*-butyl protected carboxybetaine methacrylamide monomer synthesis

Adapted from previously published procedure^49^, N-[3(dimethylamino)propyl]methacrylamide (25 g, 147 mmol, 1 equiv.) was dissolved in 200 mL of dry acetonitrile under nitrogen. *Tert*-butyl bromoacetate (34 g, 176 mmol, 1.2 equiv.) was added, and left to react overnight at 50 °C. The reaction was cooled to room temperature and the white product was precipitated with 500 mL of ether, decanted, washed with 100 mL of ether 3 times and dried under a stream of nitrogen. 1H NMR (D_2_O, 600 MHz) δ: 5.7 (s, 1H), 5.5 (s, 1H), 4.3 (s, 2H), 3.6 (m, 2H), 3.4 (t, 2H), 3.3 (m, 6H), 2.1 (tt, 2H), 1.9 (s, 3H), 1.5 (s, 9H) (Fig. S12).

### General polymerization protocol

Reaction mixtures for pDMAPMA and pOEGMA homopolymers and pOEGMA-fluorescein and pCB-fluorescein copolymers were prepared for reactions using a RAFT polymerization technique with appropriate amounts of monomer, 4-Cyano-4-(phenylcarbonothioylthio)pentanoic acid chain transfer agent (CTA), 4,4’-azobis(4-cyanopentanoic acid) initiator and solvent as detailed in Table S2. Reaction mixtures were then degassed by 3 rounds of the freeze pump thaw method, and incubated, with stirring, at 70 °C overnight. The crude polymer mixtures were then aminolysed to produce a terminal thiol by incubation with butylamine (10 × CTA mol amount) for 2 h, at pH 10, then TCEP was added, and the pH of the mixture lowered to 5 and incubated for 2 h, and finally dialyzed for 3 d against pH 5 water, and lyophilized. Synthesized pDMAPMA was then reacted with *tert*-butyl bromo acetate at a 3:1 molar excess of TBu to DMAPMA monomer content, in acetonitrile for 3 d, and dialyzed against methanol for 3 d to produce pCB-TBu. pCB-TBu was then deprotected by incubation at 50 °C for 6 h in pH 1.3 HCl, and dialyzed for 3 d against pH 5 water to yield pCB-COOH. Protection and deprotection steps were quantified by H NMR (Fig. S13). Terminal thiol presence was verified by an Ellman’s assay kit following the manufacturer protocol where thiol terminal 2mer pOEGMA (4, 40 and 400 mg mL^-1^) was incubated with Ellman’s reagent for 15 min, and quantified by absorbance measurement at 412 nm (Fig. S14).

### Polymer characterization by GPC

Characteristic molecular weights (M_n_ and M_w_) and dispersities (Ɖ) were measured by an Agilent 1260 infinity II GPC system equipped with an Agilent 1260 infinity RI detector at 30 °C, a Superose 6 Increase 10/300 GL column, and with PBS running buffer supplemented with 0.05% sodium azide at room temperature. The column was calibrated using polyethylene glycol (PEG) standards (Mn of 3000 to 60,000). Degree of polymerization (*N*) was calculated using monomer molecular weight and reported measured M_n_ by GPC (Table S3).

### Polymer grafting procedure

PDMS discs were plasma oxidized for 45 s on “high” setting, then immediately placed into 1% (v/v) APTES in dry toluene and shaken for 1 h. The APTES solution was then removed, and the discs were rinsed 3 times with dry toluene. A solution of 2 mg mL^-1^ SMCC in PBS was added to the discs and shaken for 2 h. The SMCC solution was then removed, then discs were dried and either deswelled prior to polymer grafting, reswollen with EtAc or kept swollen in toluene. The discs were then incubated with the appropriate thiol terminated polymer at 2 mg mL^−1^ in either MES or GHCl buffer at pH 6.5 for 4 d with shaking. Materials that had polymer grafting in the swollen state were then deswelled overnight. Finally, discs were incubated overnight with shaking in pH 9.3 borate buffer to hydrolyze remaining maleimides on the material surface.

### Excess maleimide assay

Maleimides on the surface of the PDMS discs were quantified using a modified fluorescence detection assay kit. Fluorescent maleimide reactive probes were prepared according to the manufacturer guidelines and 100 μL of fluorescent probe in supplied Assay Buffer was added to a well containing a PDMS disc to be assayed and incubated at room temperature overnight, with shaking. Each disc was then rinsed 3 times with DI water, dried with a laboratory wipe, and surface fluorescence was quantified by fluorescence microscopy using a Biotek Cytation5 plate reader equipped with a GFP channel filter cube.

### Fluorescent polymer content characterization

Fluorescent polymer distribution into modified elastomers was characterized by confocal laser scanning microscopy, with depth profiles Z-stacks in the channel corresponding to the fluorescein tagged copolymers. The Z-stacks were acquired at a step size of 10 µm.

### Characterization of surface hydrophilicity

Material hydrophilicity was characterized by static water contact angle measurements, and advancing and receding water contact angles (OCA 20 contact angle goniometer, with SCA 20 software). For static water contact angle, droplets of MilliQ water (2 μL, resistivity > 18.2 MΩ cm) were placed onto modified PDMS discs and photographed. One measurement per disc was made, replicates represent three separate discs. For advancing and receding measurements, MilliQ water (5 μL, resistivity > 18.2 MΩ cm) was placed onto the surfaces, and without removing the needle from the droplet, water was added or removed until the contact angle stabilized. One measurement per disc was made, on 3 different discs.

### Bacterial adhesion assay

A culture of *E. coli* BL21 was inoculated in LB broth and incubated overnight at room temperate with shaking. The following day, the culture was subcultured and grown to an OD of 0.5 and then 200 μL of this suspension was added to functionalized PDMS discs in a 96 well plate and incubated at room temperature overnight with shaking. The PDMS discs were then removed from the bacterial suspension, rinsed 3 times with sterile LB broth and placed into fresh LB broth to grown overnight. Following overnight incubation, the OD of the LB broth which had incubated overnight with the rinsed discs, was measured.

### Macrophage adhesion

PDMS discs were immobilized into a 96 well plate with PDMS (Sylgard™ 184) and cured at 80 °C for 30 min, then sterilized by incubation with 70% ethanol for 1 h, and rinsed with sterile DI water 3 times. Sterilized materials were then incubated with 100% aged FBS overnight at 37 °C at 5% CO_2_ then the serum was removed and the materials were incubated with RAW 264.7 macrophages (10 000 cells per well) for 48 h 37 °C at 5% CO_2_. Following incubation, the cell containing media was removed from the wells, surfaces were gently rinsed a single time with PBS to remove non-adhered cells from the well, and the materials were stained with Hoescht according to the manufacturer protocol prior to imaging with a Biotek Cytation5 microscope. Quantification of adhered cells was performed manually by counting in ImageJ.

### SEM characterization

Samples for SEM imaging were prepared by coating with 5 nm of platinum and attached to imaging stubs with nickel paint and carbon tape. All images were collected with a JEOL-JSM 7000F ((JEOL USA, Inc., Peabody, MA, USA), with working distances of 4.0 to 4.7 mm, and an accelerating voltage of 5 kV.

### Statistical analysis

All statistical analyses were performed using GraphPad Prism 8. Significant differences were determined by multiple comparisons corrected multiple t-tests, using the Holm-Sidak method. Significant p-values are indicated on graphs as follows *p* < 0.05 is indicated by *, *p* < 0.01 by **, and *p* < 0.001 by ***.

### Supplementary Information

Supplementary Information.

## Data Availability

The datasets generated and analysed during the current study are available from the corresponding author on reasonable request.
