# Supplementary material for: Controlled swelling of biomaterial devices for improved antifouling polymer coatings
Source: Sci Rep. 2023 Nov 15;13:19950. doi: 10.1038/s41598-023-47192-8 (PMC10651925; doi:10.1038/s41598-023-47192-8)
Supplement: Supplementary file 1 — Supplementary Information. [file 41598_2023_47192_MOESM1_ESM.pdf]

**Supporting Information**

# Grafting polymers to solvent swelled PDMS elastomers increases thin film density and antifouling performance

*Alexander H. Jesmer, April S.T. Marple, Ryan G. Wylie**

Figure S1. Surface fluorescence of pOEGMA on PDMS. 2
Figure S2. Maleimide content of elastomers after polymer grafting. 3
Figure S3. pCB-TBu ester deprotection in pH 1.3 HCl. 4
Figure S4. Grafting salt choice and concentration modifies grafted pCB-COOH content. 5
Figure S5. Apparent molecular weight of pCB changes with GHCl concentration. 6
Figure S6. Macrophage adhesion to PDMS modified with pCB-COOH. 7
Figure S7. Macrophage adhesion to PDMS modified with non-antifouling polymers. 8
Figure S8. Representative fluorescence micrographs of RAW 264.7 macrophage adhesion on polymer modified PDMS. Scale bar = 1000 µm. 9
Figure S9. Water contact angle measurements of polymer modified PDMS. 10
Figure S10. Contact angle measurements with cell maintenance media of 8mer pOEGMA PDMS. 11
Figure S11. Water contact angle measurements of pCB-*co*-APMA grafted PDMS that is crosslinked with EDC immediately following grafting and dynamic water contact angle measurements of 8mer 100 kDa pOEGMA. 12
Figure S12. H NMR spectroscopy of CB-TBu monomer. 13
Figure S13. NMR spectroscopic characterization of pCB-TBu synthesis from pDMAPMA. 14
Figure S14. Terminal thiol presence verification by Ellman assay. 15

Table S1: Polymerization conditions used for the preparation of RAFT polymer library. 16
Table S2: Calculated molecular weights, dispersities and degrees of polymerization of polymers used. 17

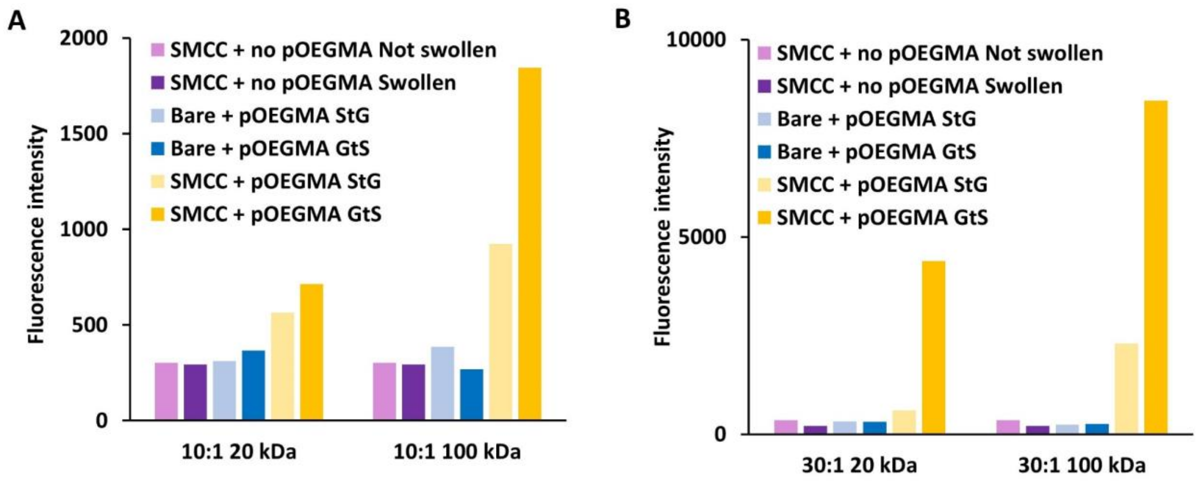


**Figure S1. Surface fluorescence of pOEGMA on PDMS.**

Grafting of fluorescent (A) 20 and (B) 100 kDa 8mer pOEGMA to PDMS with and without maleimide functionalization.

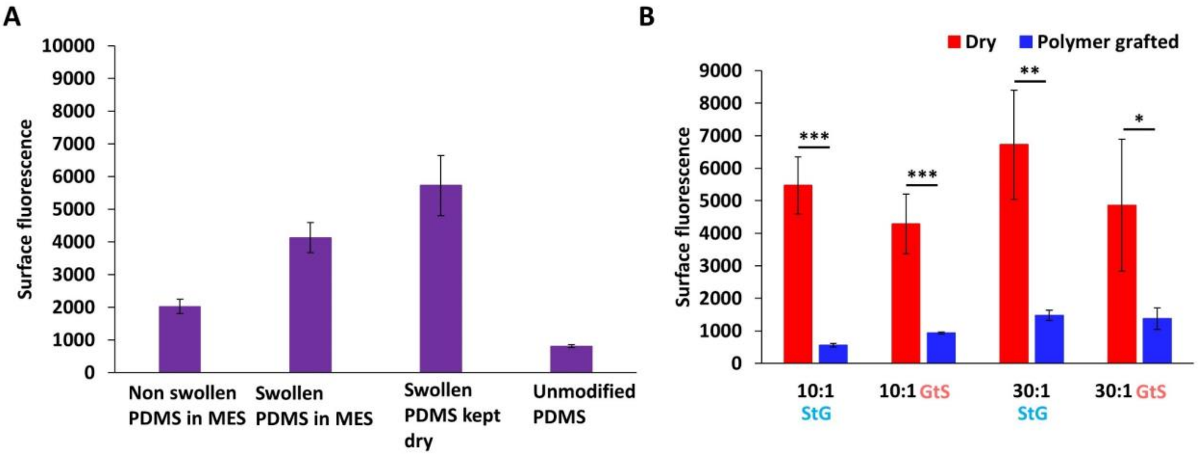


**Figure S2. Maleimide content of elastomers after polymer grafting.** (A) Surface fluorescence of SMCC modified elastomers after reaction with a thiol-fluorescein tracer. (B) Maleimide content of elastomers before and after polymer grafting. Means ± SD, n = 3.

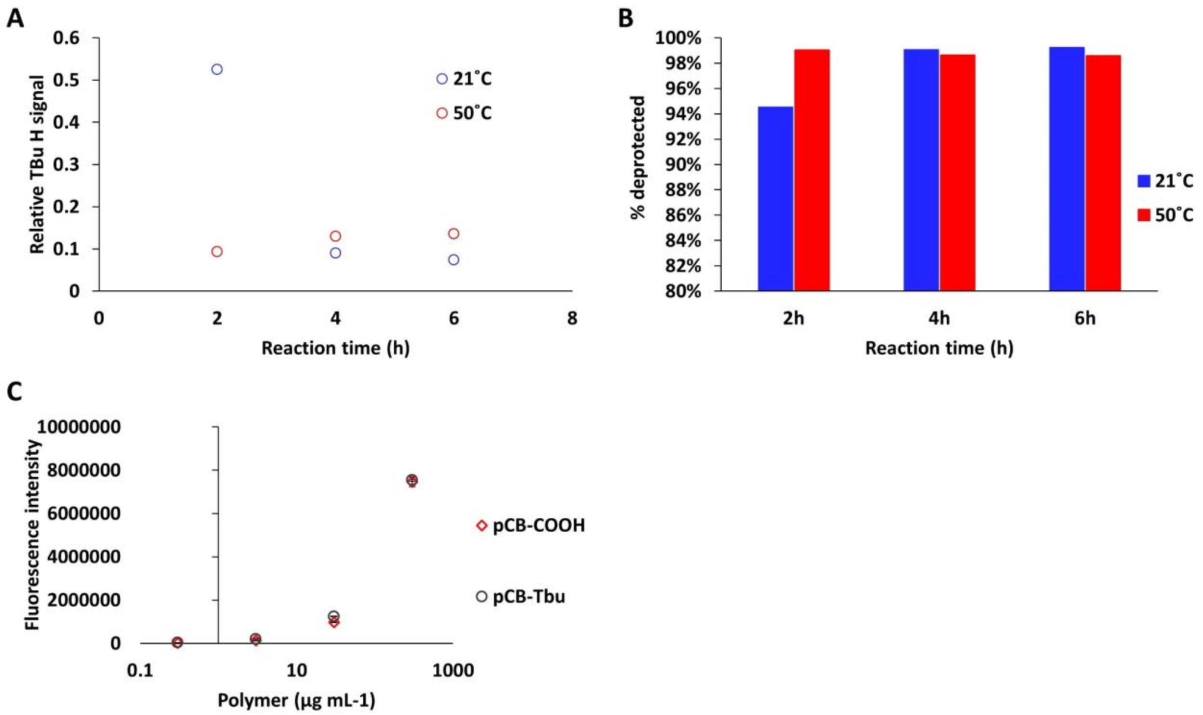


**Figure S3. pCB-TBu ester deprotection in pH 1.3 HCl.** (A) Relative *tert*-butyl group signal by NMR after exposure to HCl at pH 1.3 for between 2 and 6 hours at room temperature and 50°C. (B) Calculated percent of ester deprotection based on relative signal from NMR of pCB-TBu. (C) Solution fluorescence intensity of pCB-TBu and pCB-COOH fluorescein methacrylate copolymers. Mean ± SD, n = 3.

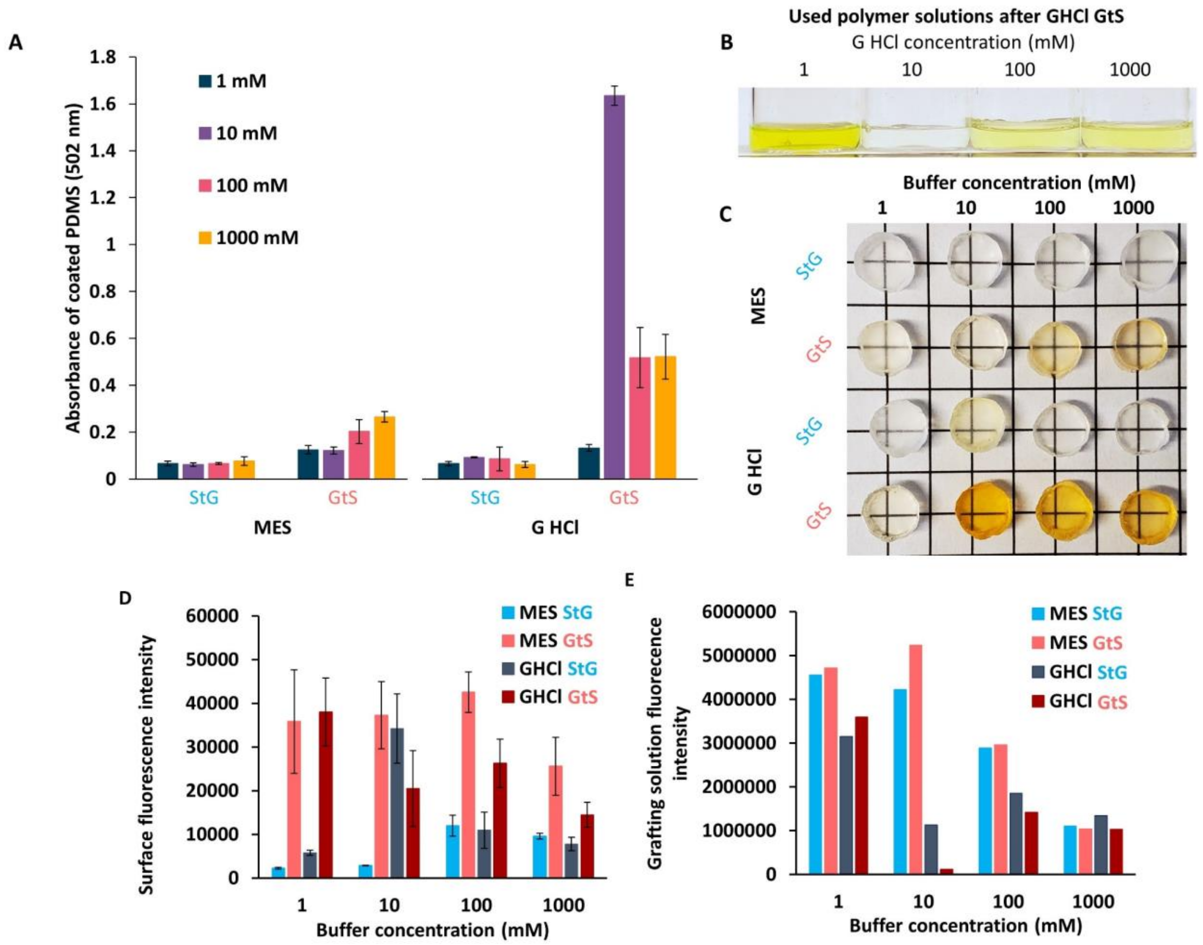


**Figure S4. Grafting salt choice and concentration modifies grafted pCB-COOH content.** (A) Absorbance of elastomers at 502 nm modified with fluorescent pCB-COOH$_f$ copolymers (mean ± SD, n = 3). Concentrations refer to MES or GHCl, polymer concentration was 2 mg mL$^{-1}$ for all cases. (B, C) Photographs of grafting solution after polymer grafting procedure and 10:1 Graft then shrink elastomers grafted with fluorescent pCB-COOH$_f$ copolymers in various buffers. (D) Surface fluorescence of 10:1 PDMS elastomers modified with fluorescent pCB-COOH in MES and GHCl grafting buffers between 1 and 1000 mM. (E) Grafting solution fluorescence of 10:1 PDMS elastomers modified with fluorescent pCB in MES and GHCl grafting buffers between 1 and 1000 mM.

To explore the influence of GHCl on pCB-COOH, we conducted gel permeation chromatography (GPC) studies in buffers containing GHCl. GPC analysis of pCB-COOH in varying GHCl buffer strengths between 1 and 100 mM (pH 6.5) showed decreasing apparent $M_w$s and hydrodynamic radii with increasing buffer concentration, while PEG standards eluted at nearly identical times, with no change in apparent $M_w$, in all three GHCl buffer strengths tested (**Figure S5**). Differences in grafting efficiency may be the result of improved polymer packing at 10 mM over 1 mM and improved thiol accessibility and reactivity at 10 mM over 100 mM due to the more extended polymer conformation at 10 mM. Guanidine has previously shown effects on amphiphilic block copolymer grafting density[42], and has also been shown to control the collapsed and uncollapsed state of elastin like peptides in solution through interactions with amide bonds[39], which are also present in pCB-COOH. Therefore, GHCl is most likely influencing the hydrodynamic radius of pCB-COOH, indicating that buffer conditions beyond solubility can also influence grafting density for zwitterionic polymers.

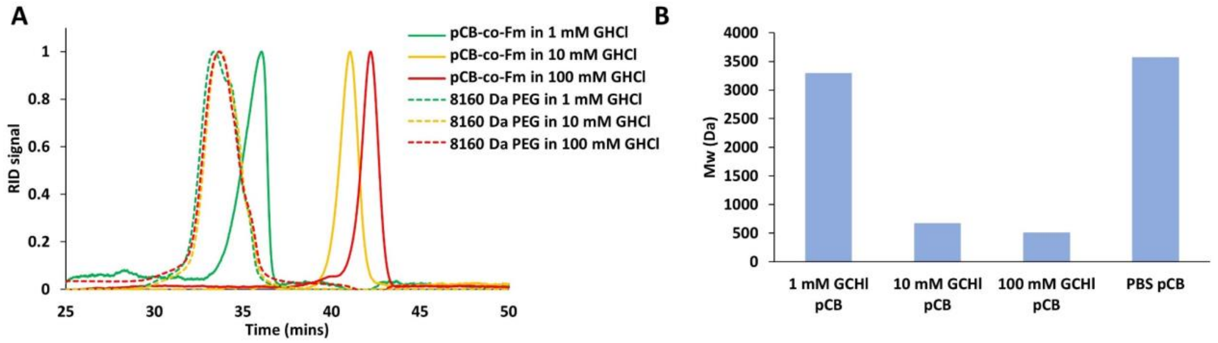


**Figure S5. Apparent molecular weight of pCB changes with GHCl concentration.** (A) GPC of PEG standards and pCB-*co*-fluorescein methacrylate in three GHCl buffer concentrations. (B) Plotted apparent molecular weight of pCB as calculated by GPC calibrated with PEG standards in PBS.

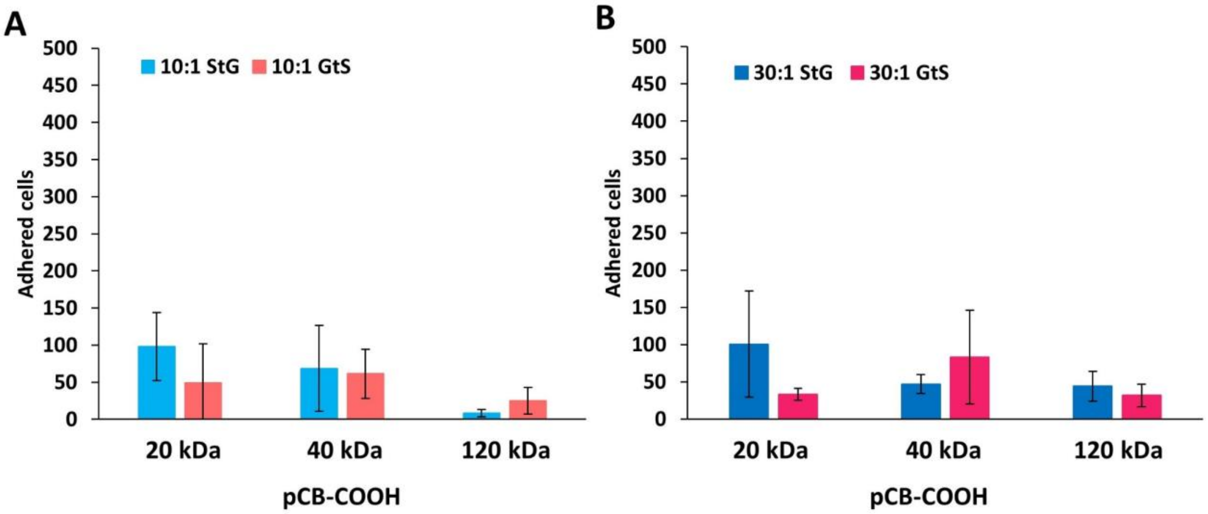


**Figure S6. Macrophage adhesion to PDMS modified with pCB-COOH.** Cell adhesion of Raw 264.7 macrophages on (A) 10:1 and (B) 30:1 PDMS modified with 20, 50, and 100 kDa pCB-COOH (mean ± SD, n = 3).

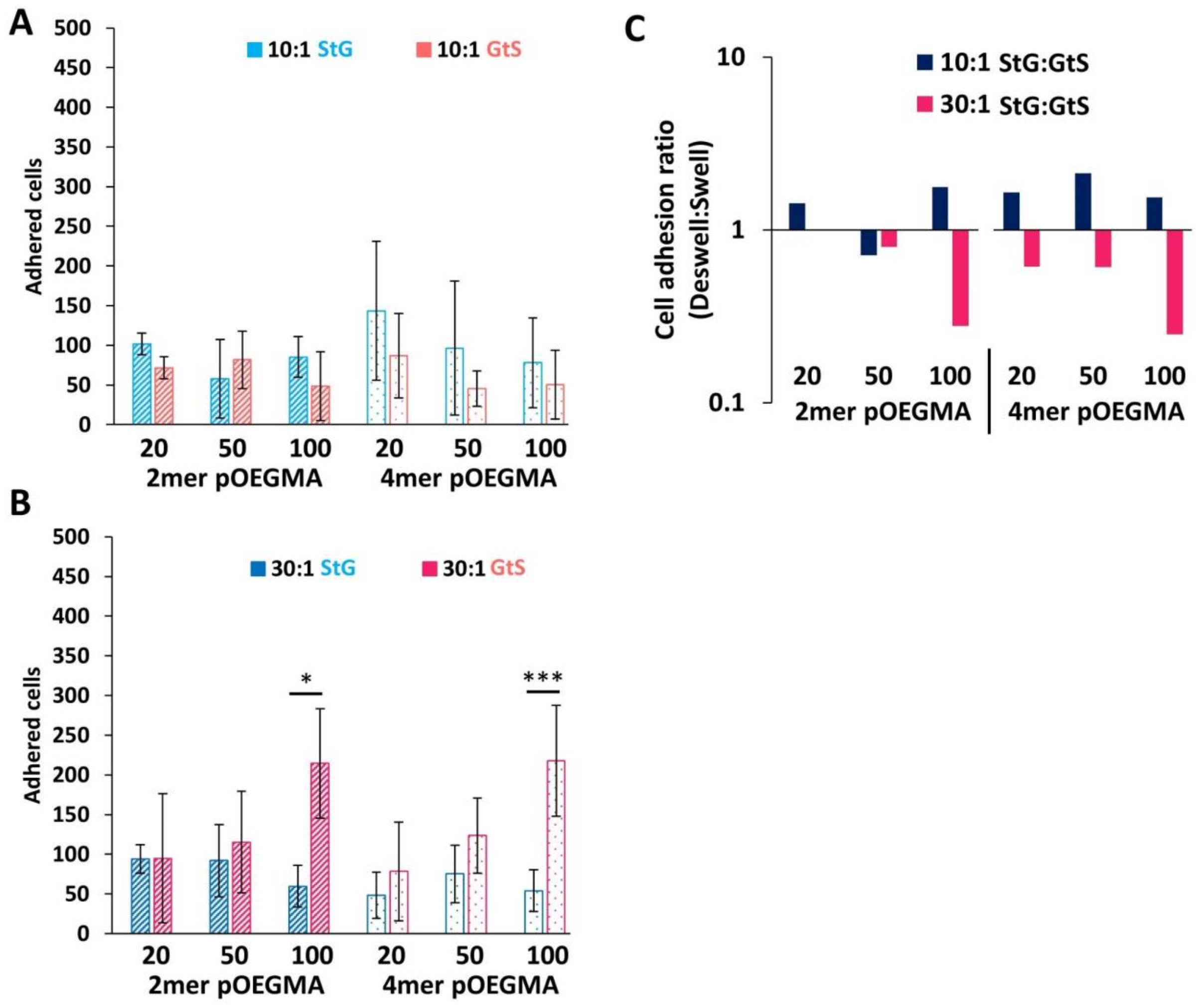


**Figure S7. Macrophage adhesion to PDMS modified with non-antifouling polymers.** (A) Cell adhesion of Raw 264.7 macrophages on 10:1 and 30:1 PDMS modified with 2mer and 4mer pOEGMA (mean ± SD, n = 3). (C) Ratio of cells adhered between Graft then shrink and Shrink then graft materials modified with 2mer and 4mer pOEGMA.

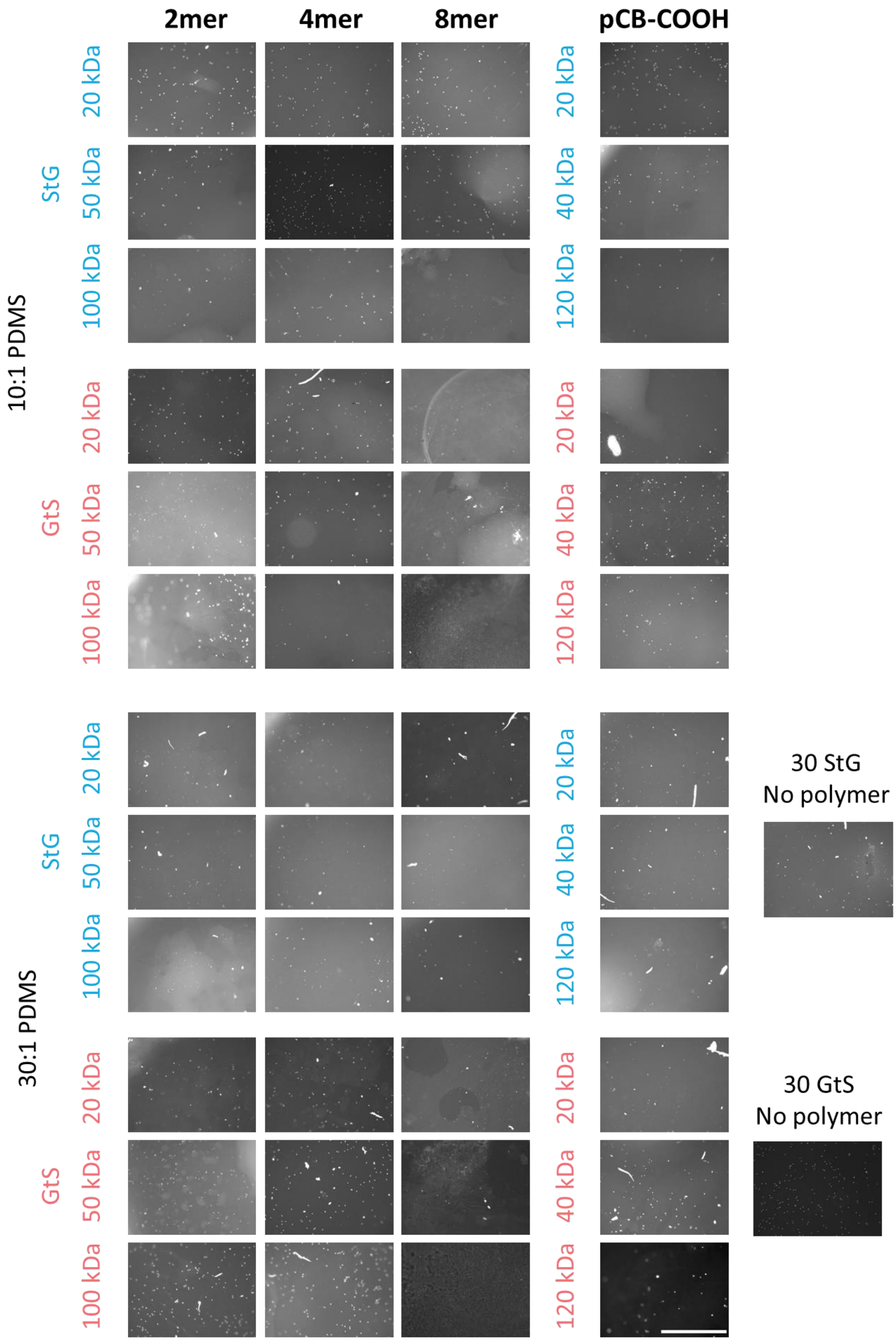


**Figure S8. Representative fluorescence micrographs of RAW 264.7 macrophage adhesion on polymer modified PDMS.** Scale bar = 1000 µm.

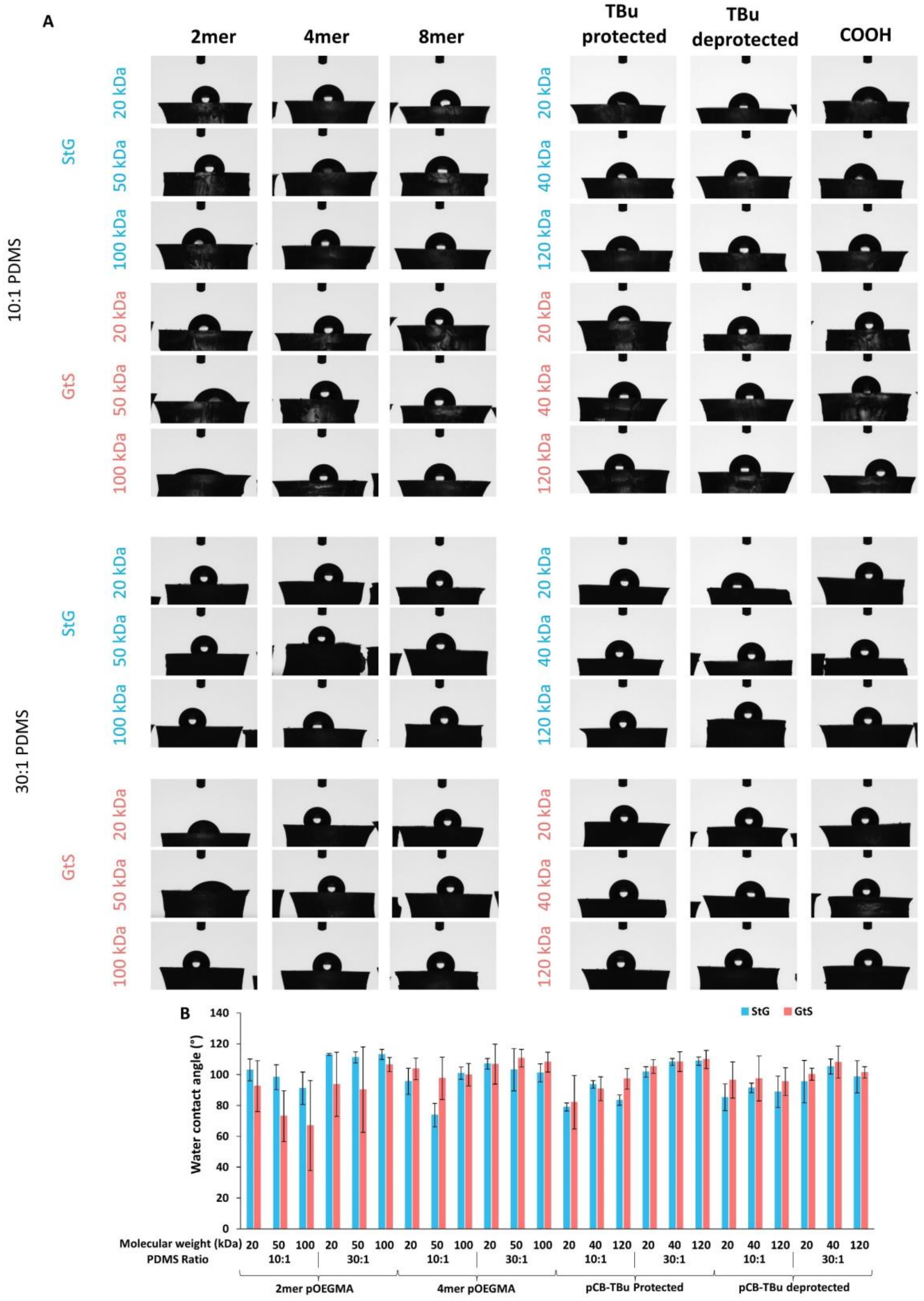


**Figure S9. Water contact angle measurements of polymer modified PDMS.** (A) Representative images of 3 µL droplets of MilliQ water on PDMS samples. (B) Average water contact angle of PDMS samples. Mean ± SD, n = 4.

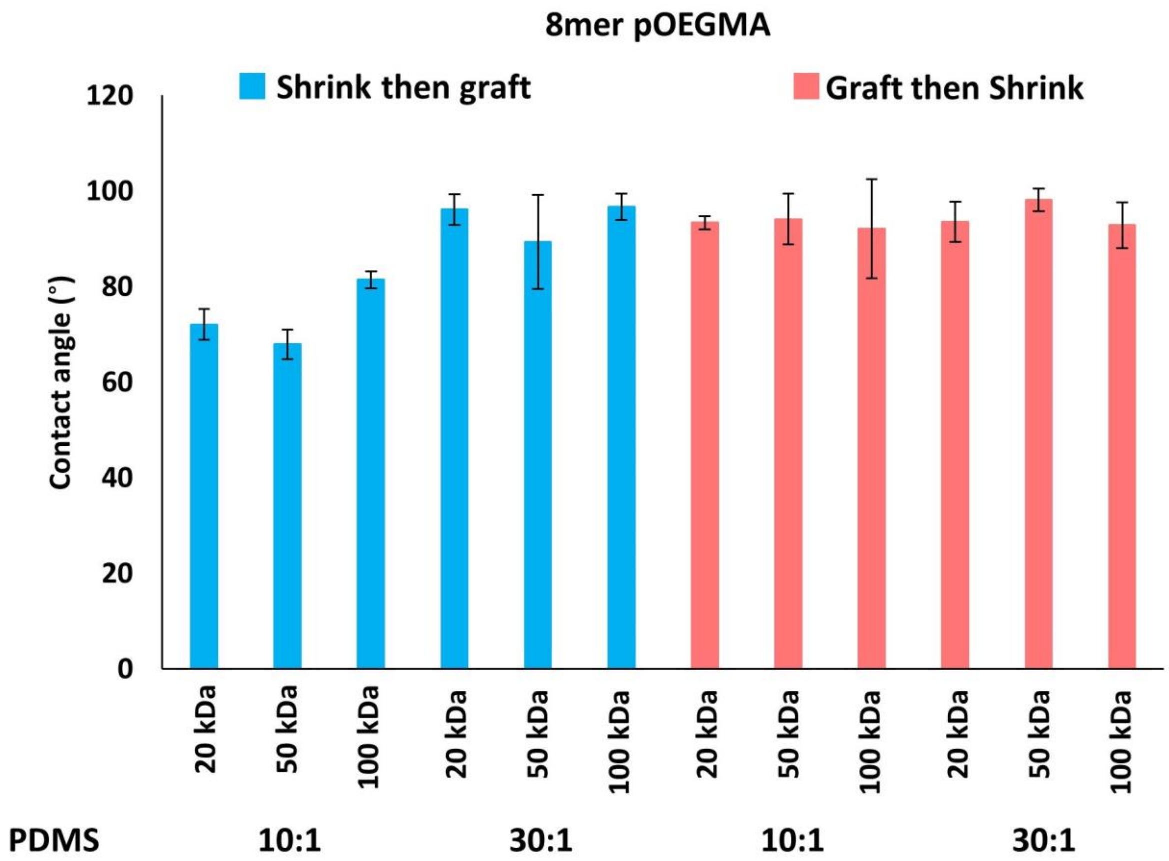


**Figure S10. Contact angle measurements with cell maintenance media of 8mer pOEGMA PDMS.** Average water contact angle on PDMS samples of 3 µL droplets of 10% FBS supplemented cell media. Mean ± SD, n = 4.

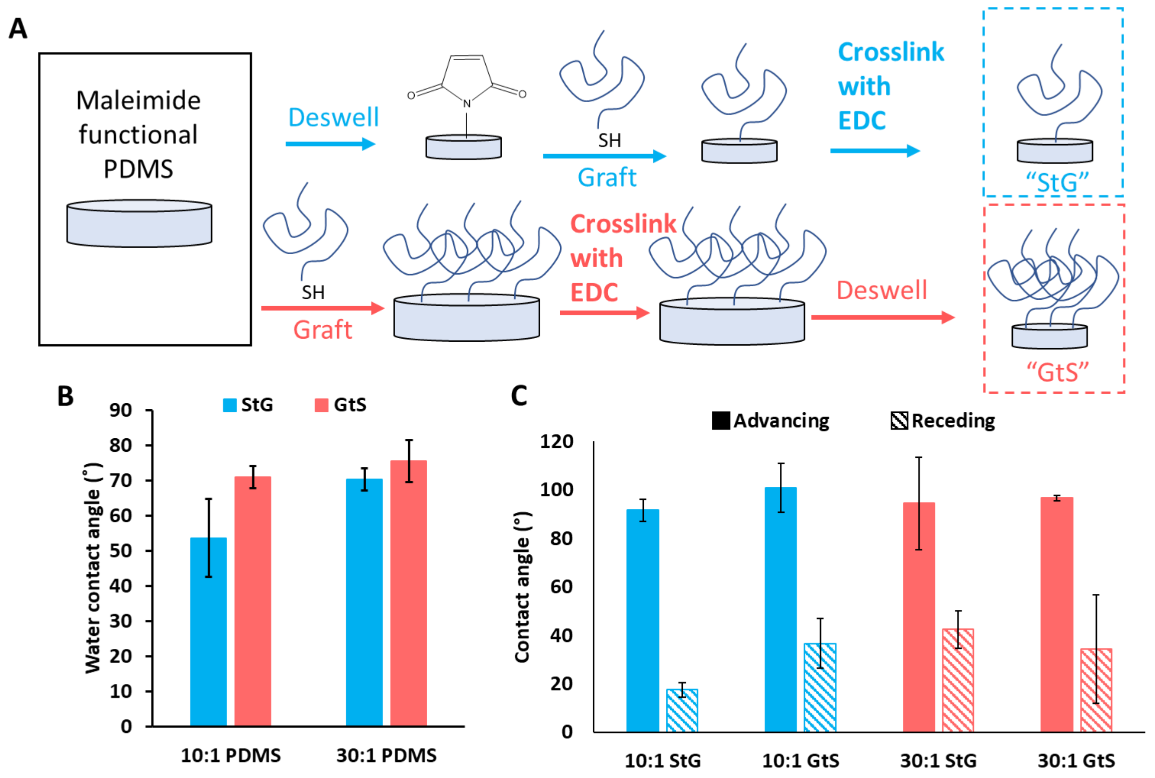


**Figure S11. Water contact angle measurements of pCB-*co*-APMA grafted PDMS that is crosslinked with EDC immediately following grafting and dynamic water contact angle measurements of 8mer 100 kDa pOEGMA.** (A) Schematic showing when EDC crosslinking was performed in the grafting and swelling process. (B) Average water contact angle on PDMS grafted with pCB-*co*-APMA, samples of 3 µL droplets of water. (C) Average advancing and receding water contact angles on 8mer pOEGMA coated PDMS samples, with ~ 5 uL droplets. Mean ± SD, n = 3.

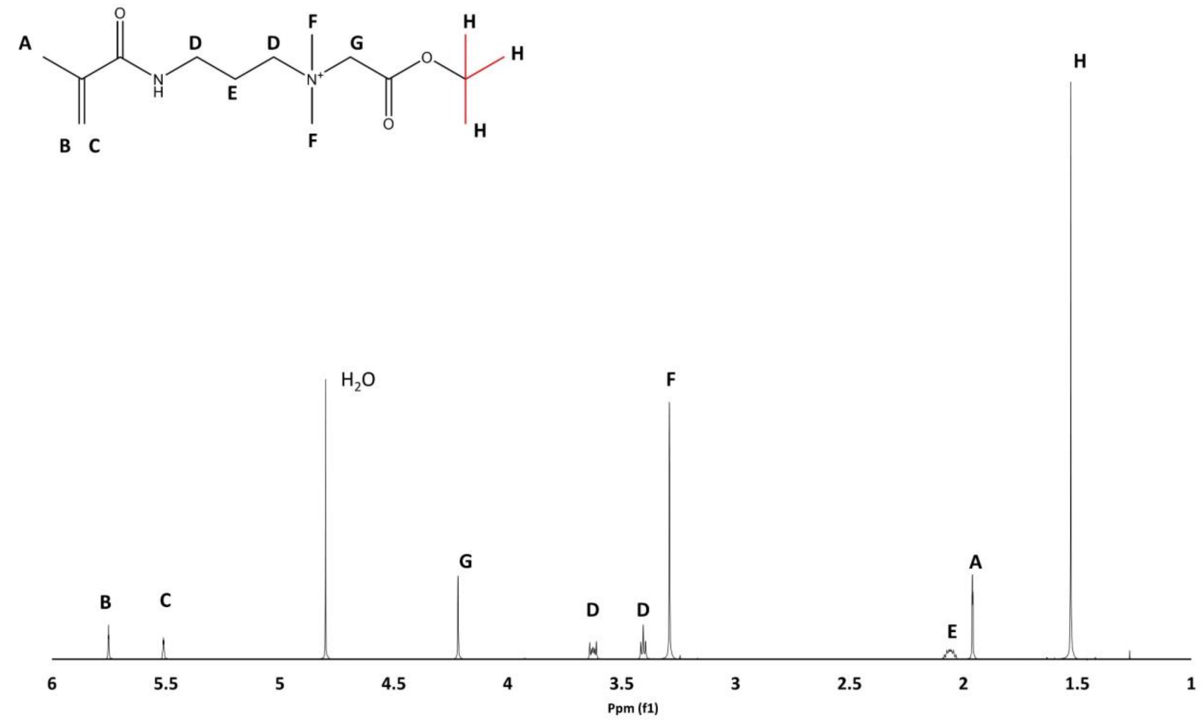


**Figure S12. H NMR spectroscopy of CB-TBu monomer.**

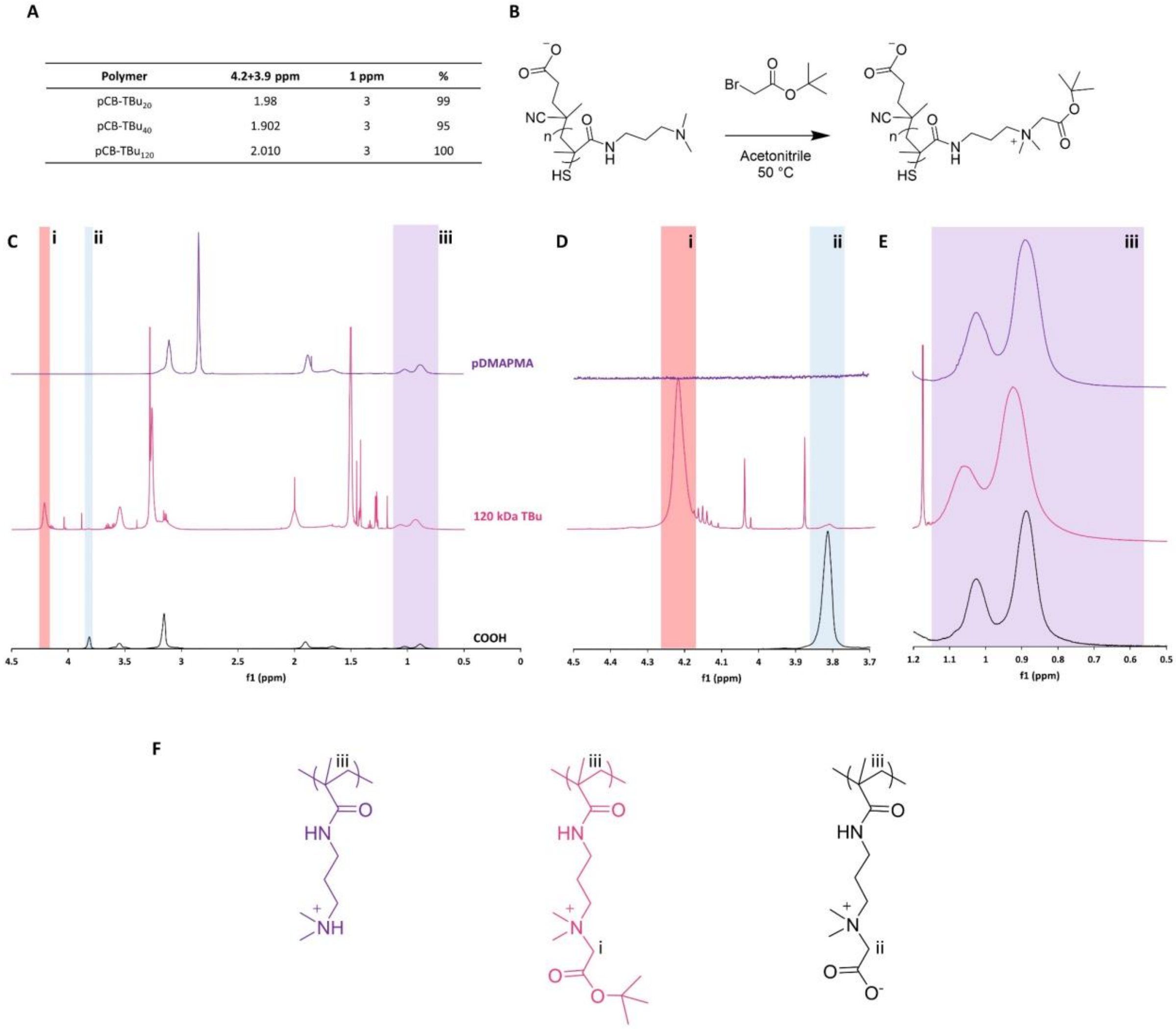

| Polymer | 4.2+3.9 ppm | 1 ppm | % |
|---|---|---|---|
| pCB-TBu$_{20}$ | 1.98 | 3 | 99 |
| pCB-TBu$_{40}$ | 1.902 | 3 | 95 |
| pCB-TBu$_{120}$ | 2.010 | 3 | 100 |



**Figure S13. NMR spectroscopic characterization of pCB-TBu synthesis from pDMAPMA.** (A) Quantification of monomer percent modification by NMR. (B) Reaction scheme of pCB-TBu preparation. (C-E) H NMR of precursor pDMAPMA, protected pCB-TBu and deprotected pCB-COOH. (F) Structures and assignments of H NMR.

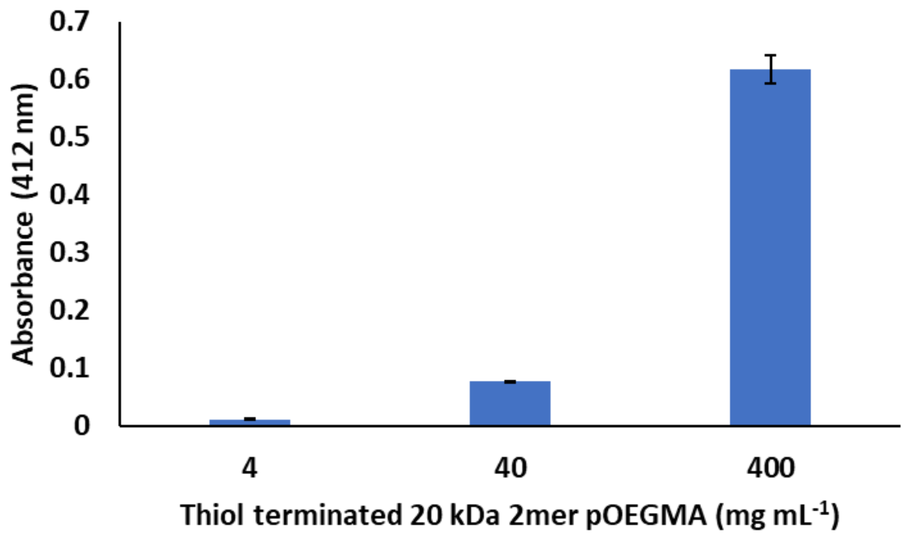


**Figure S14. Terminal thiol presence verification by Ellman assay.** Blank subtracted absorbance measurements at 412 nm of thiol terminal 2mer pOEGMA solutions following incubation for 15 minutes at room temperature with Ellman's reagent (means ± SD, n = 3).

**Table S1: Polymerization conditions used for the preparation of RAFT polymer library.**

| Monomer | Molecular weight (kDa) | Monomer (g) | CTA (mg) | Initiator (mg) | Solvent (mL) | Solvent type |
|---|---|---|---|---|---|---|
| **2mer** | 20 | 2 | 28.3 | 14.2 | 8.7 | Dioxane |
| | 50 | 2 | 11.2 | 5.6 | 8.7 | Dioxane |
| | 100 | 2 | 5.6 | 2.8 | 8.7 | Dioxane |
| **4mer** | 20 | 2 | 28.3 | 14.2 | 7.6 | Dioxane |
| | 50 | 2 | 11.2 | 5.6 | 4.7 | Dioxane |
| | 100 | 2 | 5.6 | 2.8 | 7.6 | Dioxane |
| **8mer** | 20 | 2 | 28.3 | 14.2 | 3.9 | Dioxane |
| | 50 | 2 | 11.2 | 5.6 | 3.9 | Dioxane |
| | 100 | 2 | 5.6 | 2.8 | 3.9 | Dioxane |
| **pOEGMA$_{8,9}$- fluo** | 20 | 0.5 / 0.004 | 7.1 | 3.6 | 0.98 | Dioxane |
| | 50 | 0.5 / 0.004 | 2.6 | 1.4 | 0.98 | Dioxane |
| | 100 | 0.5 / 0.004 | 1.4 | 0.7 | 0.98 | Dioxane |
| **pDMAPMA** | 20 | 2 | 27.9 | 18.7 | 11 | 2:1 Buffer*:Dioxane |
| | 40 | 2 | 14.0 | 9.3 | 11 | 2:1 Buffer*:Dioxane |
| | 120 | 2 | 4.7 | 3.1 | 11 | 2:1 Buffer*:Dioxane |
| **pDMAPMA-*co*-fluorescein methacrylate** | 50 | 1 / 0.02 | 5.7 | 2.9 | 6.6 | 2:1 Buffer*:Dioxane |
| **pCB-*co*-APMA** | 60 | 1.4 / 0.1 | 7 | 3.5 | 4 | 2:1 Buffer*:Dioxane |

*Sodium acetate buffer (pH 5, 1 M)

**Table S2: Calculated molecular weights, dispersities and degrees of polymerization of polymers used.**

| **Name** | | **Monomer MW** | **$M_n$** | **$M_w$** | **Đ** | ***N*** |
|---|---|---|---|---|---|---|
| **20 kDa** | 2mer | 186 | 5.7 | 7.8 | 1.36 | 31 |
| | 4mer | 300 | 7.9 | 9.7 | 1.21 | 26 |
| | 8mer | 500 | 7.8 | 11.1 | 1.41 | 16 |
| **50 kDa** | 2mer | 186 | 10.4 | 14.5 | 1.40 | 56 |
| | 4mer | 300 | 16.2 | 27.2 | 1.67 | 54 |
| | 8mer | 500 | 16.9 | 34.5 | 2.04 | 34 |
| **100 kDa** | 2mer | 186 | 15.9 | 30.2 | 1.90 | 85 |
| | 4mer | 300 | 44.8 | 112.3 | 2.50 | 149 |
| | 8mer | 500 | 51.3 | 157.8 | 3.07 | 103 |
| **pOEGMA$_{8,9}$- fluo** | 20 kDa | 499 | 13.1 | 14.4 | 1.10 | 26 |
| | 50 kDa | 499 | 24.4 | 34.3 | 1.41 | 49 |
| | 100 kDa | 499 | 41.1 | 84.5 | 2.06 | 82 |
| **pCB-TBu** | 20 kDa | 285 | 16.0 | 23.5 | 1.46 | 56 |
| | 40 kDa | 285 | 33.1 | 83.9 | 2.53 | 116 |
| | 120 kDa | 285 | 122.1 | 409.1 | 3.34 | 428 |
